# Supplementary material for: Telomere attrition becomes an instrument for clonal selection in aging hematopoiesis and leukemogenesis
Source: Nat Genet. 2025 Aug 28;57(9):2215–25. doi: 10.1038/s41588-025-02296-x (PMC12425810; doi:10.1038/s41588-025-02296-x)
Supplement: Supplementary file 1 — Supplementary Figs. 1–5, Methods and Notes 1–2. [file 41588_2025_2296_MOESM1_ESM.pdf]

nature genetics

Supplementary information https://doi.org/10.1038/s41588-025-02296-x

Telomere attrition becomes an instrument for clonal selection in aging hematopoiesis and leukemogenesis

In the format provided by the authors and unedited

Table of Contents

Supplementary Figures **2**

Supplementary Methods *7*

Mutation calling and variant filtering 7

Supplementary Note 1: Analysis of phylogenetic trees **9**

Supplementary Note 2: Analysis of TERT promoter mutations in UK Biobank Whole Genomes **11**

Data exploration and cleaning 11

Association with polygenic risk score for leukocyte telomere length 21

Age-related prevalence of TERTp putative drivers versus splicing mutations 22

Association between TERTp variants and lymphocyte count 25

Supplementary References **28**

Supplementary Figures

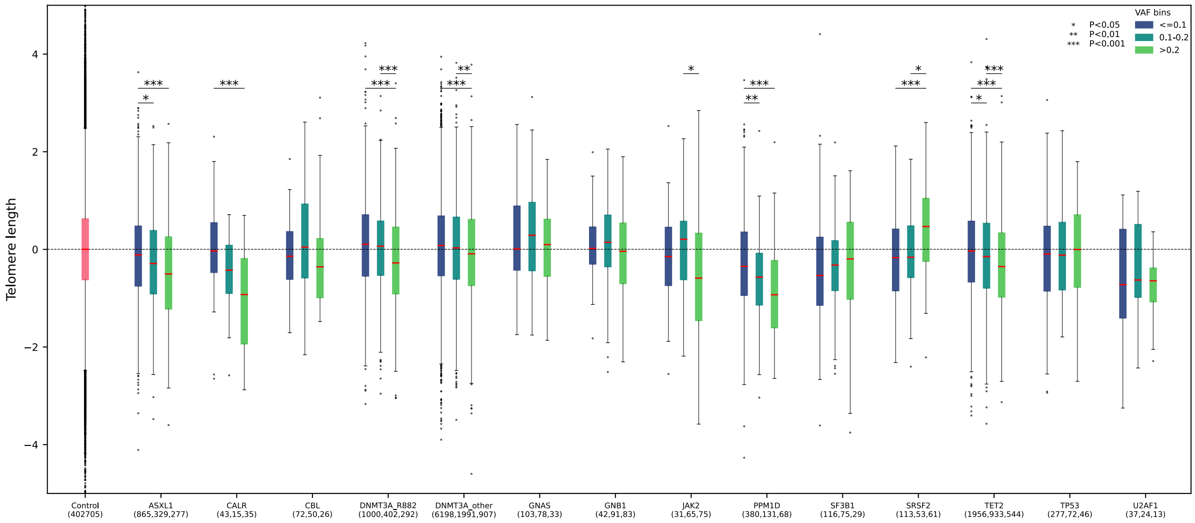

Supplementary Fig. 1. Impact of increasing VAF on LTL by CH subtype.

Boxplots showing variation in measured telomere length by CH subtype and clonal size. Telomere length was adjusted for several covariates (sex, age, smoking status, genetic principal components from 1 to 10, WBC counts and percentages of types of WBC) using multiple variable regression. Individuals within each CH subgroup were divided into those with VAF<0.1 (dark blue), VAF 0.1-0.2 (turquoise), and VAF>0.2 (green). Red lines and boxes mark the median and interquartile range, respectively. Whiskers extend to the lowest and highest data points within Q1-1.5×(Q3-Q1) and Q3+1.5×(Q3-Q1) where Q1 and Q3 represent the first and third quartiles, respectively. The control group includes all participants without any CH mutation. Number of mutation carriers in each group are shown in brackets below the gene names. Horizontal bars and “*” indicates groups with significant differences (adjusted P < 0.05) in LTL with P values derived by pairwise two-sided Wilcoxon Rank Sum tests with Bonferroni correction for multiple testing within each CH subtype. *** P < 0.001, ** P < 0.01, * P < 0.05. Y-axis is limited to the range [-5,5] to allow for better visualization of LTL differences.

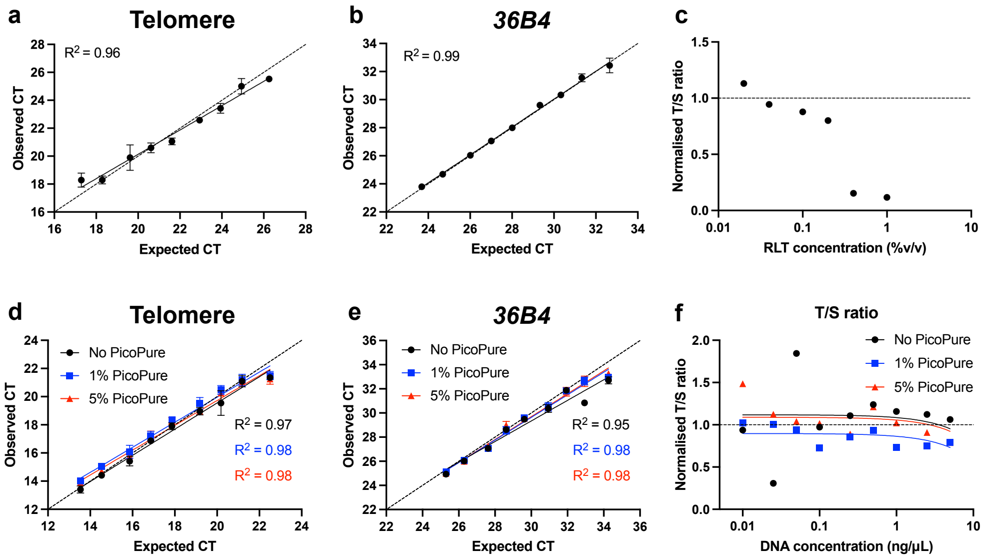

Supplementary Fig. 2. Telomere qPCR accurately estimates telomere length across a range of DNA concentrations.

a,b, Observed and expected CT values for telomere and a single copy gene (*36B4*) across a range of input DNA amounts (0.01-5ng) under standard conditions. **c**, Change in T/S ratio with increasing concentrations of RLT lysis buffer in the reaction. **d-f**, Performance of telomere qPCR across a range of DNA input amounts (0.01-5ng) and several concentrations of PicoPure lysis buffer assessed by observed and expected CT (**d,e**) and normalized T/S ratio (**f**). Each qPCR reaction was performed in triplicate (n=3) with each point showing the mean observed CT and error bars representing the mean values ± standard deviation of CT value. Error bars are not shown when the mean values ± standard deviation of CT values did not exceed the width of the point. Expected CT values were calculated with reference to the observed CT at 0.5ng input and assuming a two-fold change in signal per cycle. R^2 values and lines of best fit were calculated using a simple linear regression model.

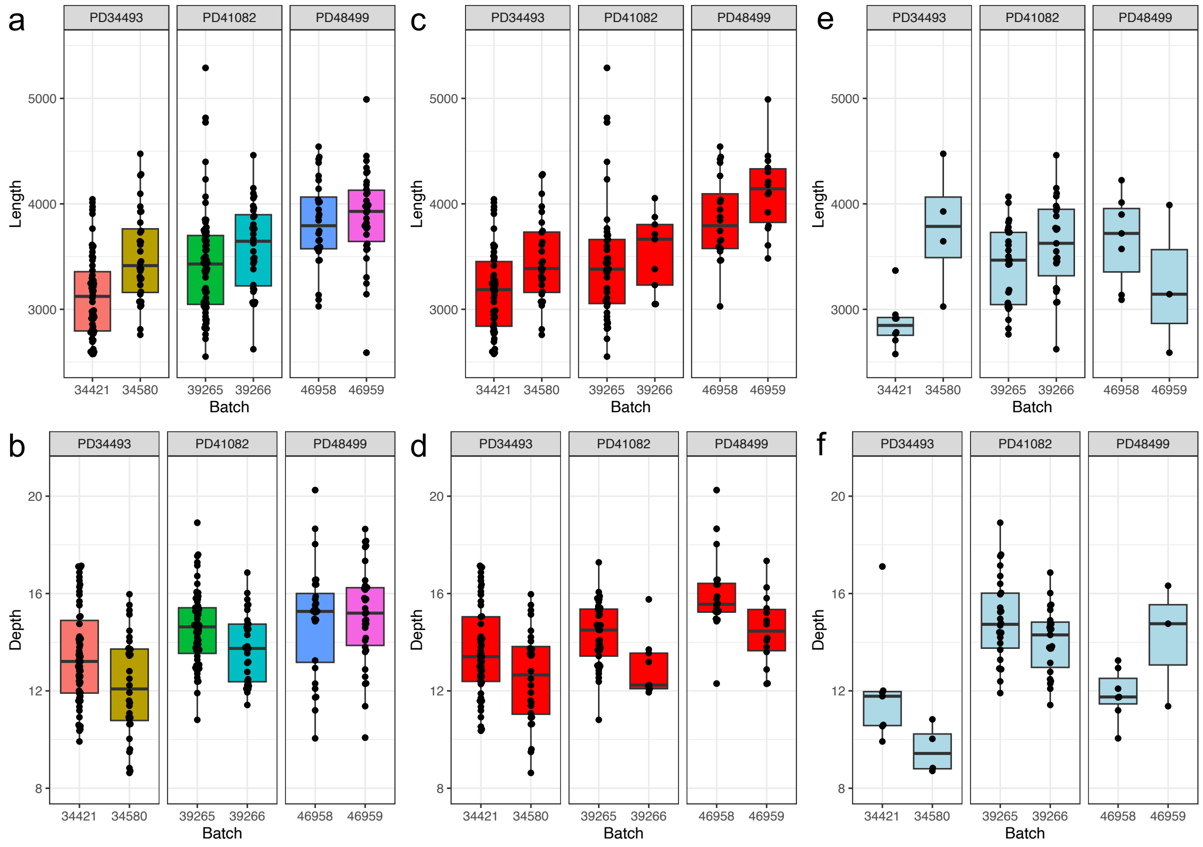

Supplementary Fig. 3. Confounders of NGS-estimated telomere length

a,b, Variation in NGS-estimated telomere length (a) and sequencing depth (b) across batches and individuals use in phylogenetic analyses. **c-d**, Variation in NGS-estimated telomere length (c) and sequencing depth (d), across red (erythroid) colonies only. **e,f**, Variation in length (e) and depth (f) in white (non-erythroid) colonies only.

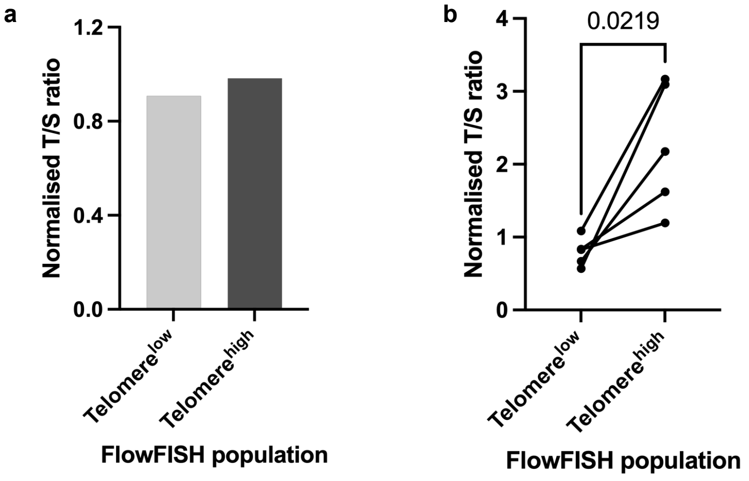

Supplementary Fig. 4. Telomere qPCR confirms differences in telomere length between flow-FISH-sorted populations.

a, Telomere qPCR from telomere^{low} (≤33rd percentile telomere length) and telomere^{high} (≥66th percentile telomere length) populations from the single individual shown in Figure 4b-c. **b**, Telomere qPCR results from the telomere^{low} (<10th percentile telomere length) and telomere^{high} (>90th percentile telomere length) populations shown in Figure 4d-h (n=5). P value was calculated using a two-sided paired t test after checking that the assumptions of the test were met (t = 3.645, df = 4, P = 0.0219).

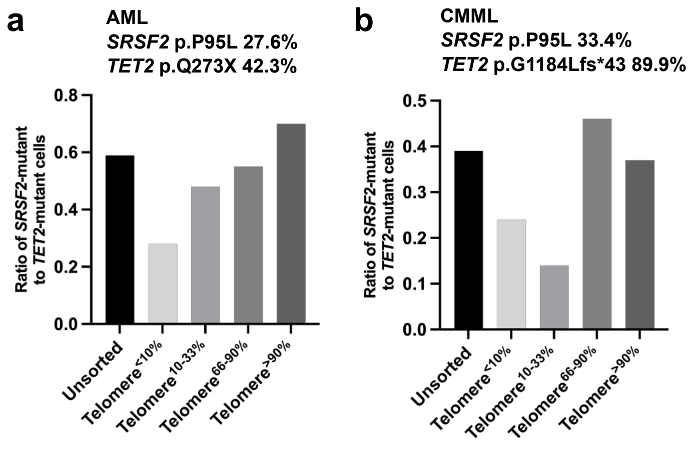

Supplementary Fig. 5. Impact of splicing factor mutations on telomere length quantified by NGS.

a-b, Ratio of *SRSF2*-mutant and *TET2*-mutant cells in PBMCs sorted by the indicated percentile telomere lengths from (a) a patient with AML and (b) a patient with CMML (VAFs quantified by NGS).

Supplementary Methods

Mutation calling and variant filtering

Somatic variants in known CH-associated genes (Supplementary Table 13) were called from whole exome sequencing data using Mutect2 (GATK v. 4.1.3.0) in the tumor-only mode with default parameters. Germline variants were filtered out using panel-of-normals from the 1000 Genomes Project (1000GP) and the Genome Aggregation Database (gnomAD). Read orientation artifacts were removed using GATK's LearnReadOrientationModel and FilterMutectCalls. Along with the variants marked as "PASS" by the FIlterMutectCalls, variants initially flagged as "germline" or "weak_evidence" but marked as "PASS" in at least five other samples were also retained for CH identification. VEP (v. 102) was used to annotate the variants. The following filtering criteria were then applied: 1) at least one read supporting the alternate allele on both forward and reverse strands; 2) minimum read depth of 7 for SNVs and 10 for indels/substitutions; and 3) minor allele frequency (MAF) < 0.001 in 1000GP and gnomAD.

Final CH variant selection required: 1) presence of the variant in a list of putative CH variants (Supplementary Table 13); 2) at least three reads supporting the alternate allele in at least one sample; and 3) one of the following: a) if the variant was present in ≥10 samples, the median variant allele frequency (VAF) must be ≤0.3 and the 90th percentile of the VAF distribution ≥0.1; or b) if the variant was present in <10 samples, it must be confirmed as somatic in at least one sample by rejecting ($p<0.001$, one-sided exact binomial test) the null hypothesis that the alternate allele represents 50% of reads (95% for X-linked genes in males). *CHEK2* variants were filtered differently as described below. Variants satisfying the following conditions were selected, 1) Marked as "PASS" by Mutect2, 2) There are at least 3 reads supporting the alternate allele, 3) $P<0.001$ in the binomial test where the null hypothesis is that 50% of the reads are alternate allele 4) Variant is truncating/start lost/frameshift or splice site variant.

Power calculations

An inclusion threshold of >100 cases was chosen following power calculations performed using the "samplesizelogisticcasecontrol" package (version 2.0.2) in R. In summary: in a case-control study with a continuous predictor and binary outcome, with 100 cases and approximately 450,000 controls, we have a power of 80% to detect protective and risk odds ratios of 0.68 and 1.47, respectively, after adjusting for multiple comparisons. Given the lack of power to confidently detect and comment on subtle associations of either measured LTL or LTL-PRS with CH driven by mutations less frequent than this threshold, we excluded those CH subtypes from downstream analysis with the exception of *U2AF1* due to its recently reported association with CH in telomere biology disorders[1].

Telomere qPCR

Colony telomere length was estimated using quantitative polymerase chain reaction (qPCR)[2]. Reactions amplifying the telomere repeat region or single copy gene (*36B4*) were performed in separate 96-well plates with the position of samples consistent between plates. Each 20µL reaction contained 0.5ng of genomic DNA and 10µL of QuantiTect SYBR Green PCR Mastermix (Qiagen 204143). Telomere reactions contained 270nM tel1 and 900nM tel2 primers and were run using the following conditions: 95°C for 15 minutes, 40 cycles of (95°C for 15 seconds, 54°C for 2 minutes). *36B4* reactions contained 300nM 36B4u and 500nM 36B4d primers and were run using the following conditions: 95°C for 15 minutes, 40 cycles of (95°C for 15 seconds, 58°C for 1 minute). The mean C_T from the telomere (T) and single copy gene (S) reactions was calculated and the T/S ratio was normalized to a standard curve generated from cell line DNA. Colonies were excluded (45/96) if the measured C_T value was within 0.5 of the negative control (n = 27), as this indicated a failed reaction, or a genotype could not be confidently established by Sanger sequencing following two repeats (n = 18).

Targeted amplicon sequencing

Purified DNA from sorted populations was prepared for targeted amplicon sequencing similar to previously described methods[3,4]. 10ng of purified genomic DNA was amplified in a 25µL reaction using HiFi HotStart ReadyMix (Kapa 07958927001) and primers targeting selected regions commonly mutated in myeloid malignancy at a final concentration of approximately 4nM (adjusted for individual primer pairs to attain similar coverage between positions) and placed in a thermocycler under the following conditions: 95°C for 3 minutes, 6 cycles of (98°C for 20s, 65°C for 60s, 60°C for 60s, 55°C for 60s, 50°C for 60s, 70°C for 60s). Following this first round of PCR, samples were kept on ice (to reduce non-specific amplification) and 1µL of 10µM i5/i7 index primers were added to each reaction, mixed and placed in a thermocycler under the following conditions: 19 cycles of (98°C for 20s, 62°C for 15s, 72°C for 30s), 72°C for 60s.

Equal volumes of up to 24 samples (amplified with unique i5/i7 index primer combinations) were pooled and a 0.55-0.75X double-sided SPRI bead clean-up was performed. Libraries were quantified using a Bioanalyzer 2100 (Agilent) and sequenced at 150bp PE on MiSeq Nano. In most cases, a second SPRI-bead clean-up (0.75X left-sided) was necessary to reduce contamination of the library (300-400bp) with adaptors (180-200bp), as the latter can interfere with sequencing.

Sequenced reads were aligned to the GRCh38 human genome assembly using BWA[5] using default parameters and without marking duplicated reads. The mpileup module in Samtools 1.18[6] was used to detect mutant reads at several loci (Supplementary Table 17).

Supplementary Note 1: Analysis of phylogenetic trees

Quality control of phylogenetic trees and colony filtering
Hematopoietic colonies were filtered to exclude those with low clonality (a median variant allele fraction of <0.4, corresponding to a clonal fraction of <0.8) or low sequencing depth (mean depth <6x). Further filtering of colonies was undertaken following quality control of phylogenetic trees, where colonies with probable cross-contamination and duplicate colonies were identified and removed. The number of colonies excluded at each of these two steps (denoted “Colony QC” and “Tree QC” respectively) is summarised in Supplementary Table 11. More colonies were filtered for PD48499 than other individuals, reflecting the fact that a larger proportion of hematopoietic colonies in this individual exhibited a low clonal fraction.

Sources of confounding in NGS estimated telomere length.
We considered various aspects of hematopoietic colony generation and sequencing as potential confounders of NGS-estimated telomere length. We observed an apparent batch effect in our telomere length estimates, wherein batches (sequencing runs) with lower sequencing depth exhibited slightly higher telomere length estimates (Supplementary Fig. 3a,b). We also noted differences in telomere length estimates and sequencing depth between red (erythroid) versus white hematopoietic colonies (Supplementary Fig. 3c-f), with telomere lengths between red and white colonies exhibiting greater similarity when compared within the same batch.

To model telomere length in our cohort of 248 colonies across three individuals and two timepoints, whilst adjusting for possible confounders, we first fit a linear mixed effects model on only the driverless colonies (n = 124):

Colony telomere length ~ Age + (1 | Batch)

We then added colony type (red/white) as an additional random effect to this null model, but found that this did not significantly improve the model, as expected from the earlier observation that the apparent disparity in length estimates was attenuated when batch was taken into account. As such, we did not include this as a covariate in our null model. We subsequently fitted our null model on the entire cohort of hematopoietic colonies (n = 248), and compared this with our alternative model, which included genotype (splicing driver mutation/other driver mutation/no driver mutation) as a fixed effect:

Colony telomere length ~ Age + Genotype + (1 | Batch)

We used the ANOVA function for model selection using Bayesian Information Criteria (BIC), and in each case considered only converged, non-singular models. The comparison of our null model and alternative model demonstrated that the alternative model (including genotype as a fixed effect) was a better fit for our data:

```
## Data: final.modelling.df
## Models:
## null.model: Length ~ Age + (1 | batch)
## alternative.model: Length ~ Mutation_type + Age + (1 | batch)
##                   npar    AIC    BIC  logLik deviance  Chisq Df Pr(>Chisq)
## null.model           4 3751.4 3765.5 -1871.7   3743.4
## alternative.model    6 3718.4 3739.5 -1853.2   3706.4 37.007  2  9.206e-09 ***
## ---
## Signif. codes:  0 '***' 0.001 '**' 0.01 '*' 0.05 '.' 0.1 ' ' 1
```

We therefore selected the alternative model as our final model of colony telomere length:

```
## Linear mixed model fit by REML ['lmerMod']
## Formula: Length ~ Mutation_type + Age + (1 | batch)
##    Data: final.modelling.df
##
## REML criterion at convergence: 3670.1
##
## Scaled residuals:
##     Min      1Q  Median      3Q     Max
## -3.4521 -0.6361 -0.1710  0.5472  3.7390
##
## Random effects:
##  Groups   Name        Variance Std.Dev.
##  batch    (Intercept)  28438   168.6
##  Residual             175809   419.3
## Number of obs: 248, groups:  batch, 6
##
## Fixed effects:
##                          Estimate Std. Error t value
## (Intercept)              4592.172    443.740  10.349
## Mutation_typeOther_driver -174.412     93.183  -1.872
## Mutation_typeSplicing      298.378     64.851   4.601
## Age                        -15.940      6.322  -2.521
##
## Correlation of Fixed Effects:
##             (Intr) Mtt_O_ Mttn_S
## Mttn_typOt_  0.019
## Mttn_typSpl -0.044  0.444
## Age         -0.983 -0.076 -0.018
```

Ideally, we would have included both individual (PD34493/PD41082/PD48499) and batch as random effects in our model, but our small sample size precluded the incorporation of multiple or complex terms, and as batch is entirely nested within the "individual" variable (since batches were not shared across individuals), we elected to fit a random intercept for batch over individual. Likewise, we would ideally have fitted a random slope for genotype, but in introducing this we produced only singular models.

Independent validation of findings using qPCR measurements of telomere length

To validate our observations based on NGS-estimated telomere lengths derived using Telomerecat, we orthogonally measured telomere length by quantitative polymerase chain reaction (qPCR) in additional single HSPC colonies from PD34493 (shown in Fig. 3a and Extended Data Fig. 5). Colonies were genotyped for the presence of the *SF3B1*-K666N and *U2AF1*-Q157R driver mutations and telomere length measured as relative telomere to single copy gene (T/S) ratio[2]. We observed the same pattern of increased telomere length in splicing factor mutant versus wild-type colonies as we had observed in our NGS-based estimates of telomere length (Extended Data Fig. 6). In our qPCR estimates, which featured more *U2AF1*-mutant colonies, the difference in T/S ratio between wild-type and *U2AF1*-mutant colonies was significant (p = 0.004, Wilcoxon Rank Sum test)

Comparison of U2AF1-mutant clade with more closely related wild-type clades

We observed a non-significant increase in the mean NGS-estimated telomere length in colonies of the *U2AF1*-mutant clade by comparison with colonies lacking splicing factor gene mutations from the same individual (PD34493). As the *U2AF1*-mutation was acquired by a cell belonging to a large clade that had originated from a single HSC, we had the opportunity to assess its impact on telomere length more directly, by comparison to related cells/colonies within this large clade. For this, we sequentially compared the *U2AF1*-mutant clade with more closely related clades and found that the trend towards longer telomeres in the former was maintained and approached statistical significance (Extended Data Fig. 5).

Supplementary Note 2: Analysis of TERT promoter mutations in UK Biobank Whole Genomes

Data exploration and cleaning

Here, we explore the data generated by performing pileup at each position along the *TERT* promoter. This is the data that was generated by Sruthi Cheloor Kovilakam by running Samtools mpileup across the *TERT* promoter on WGS data in the UK Biobank.

We begin by exploring the dimensions of the table of all of these pileup calls:

```
## [1] 4688015      10
```

There are 4.68 million rows, that is, 4.68 million alternate allele calls (for 489,548 individuals - i.e. almost all individuals in the UKB). This means that across the region of interest (201bp long), each individual has on average 9-10 alternate allele calls - much too high to be true passenger/driver mutations, and already strongly suggestive of a lot of sequencing noise/error in the unfiltered calls.

We next examine the range of depths to see if low depth regions have been filtered - here we survey the deciles of the depth across all positions:

```
##   0%  10%  20%  30%  40%  50%  60%  70%  80%  90% 100%
##    2   22   26   29   31   34   37   40   43   49  242
```

We can see that the depth across all position ranges from 2-242 reads, though most sites have 22-49 reads, and the median depth is 34 reads.

Similarly, we can examine the deciles for the variant allele fraction (VAF):

```
##         0%        10%        20%        30%        40%        50%        
60%
##  0.4132231  2.0833333  2.3809524  2.6315789  2.8571429  3.1250000  3.44
82759
##        70%        80%        90%       100%
##  3.8461538  4.5454545  5.8823529 72.2222222
```

We can see that the median VAF is 3.1%, and that most alternate alleles are at low VAF (<6%), however, there are a few sites with very high VAF that might be more consistent with germline variants.

We can visualize depth and VAF across the *TERT* promoter - here, we color by whether or not the position is one of 3 sites that we propose to be driver mutations based on a priori knowledge of the positions of somatic rescue mutations in telomere biology disorders[1] (chr5:1295046:T:G, chr5:1295113:G:A, and chr5:1295135:G:A):

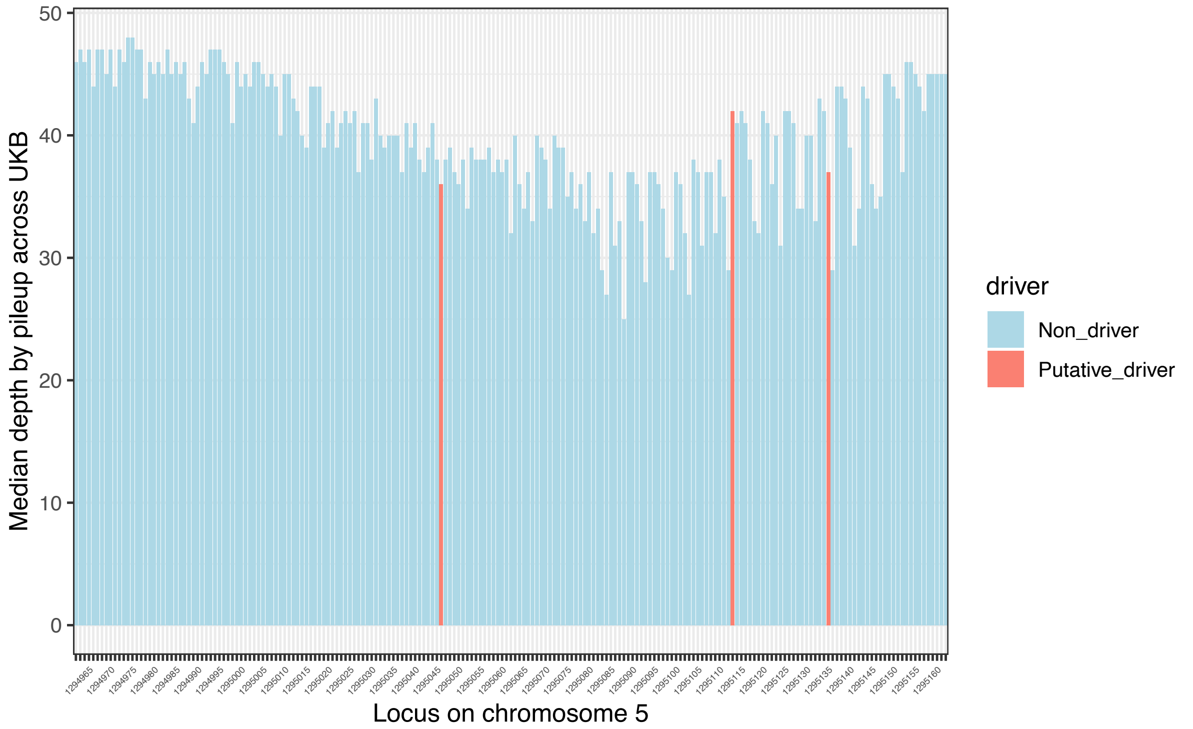

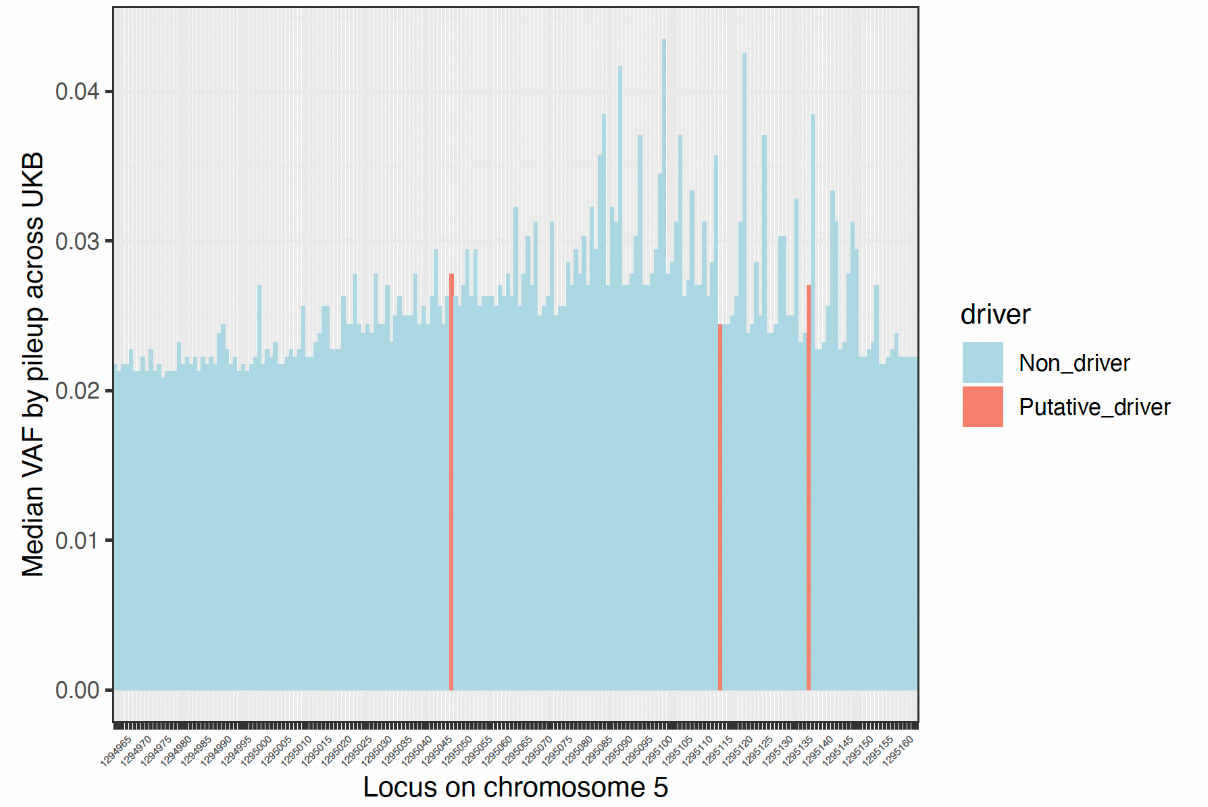

As expected, we see an inverse relationship between the median depth at each position and the median VAF of the alternate allele calls. We can more easily visualize this here by directly plotting median VAF vs median depth:

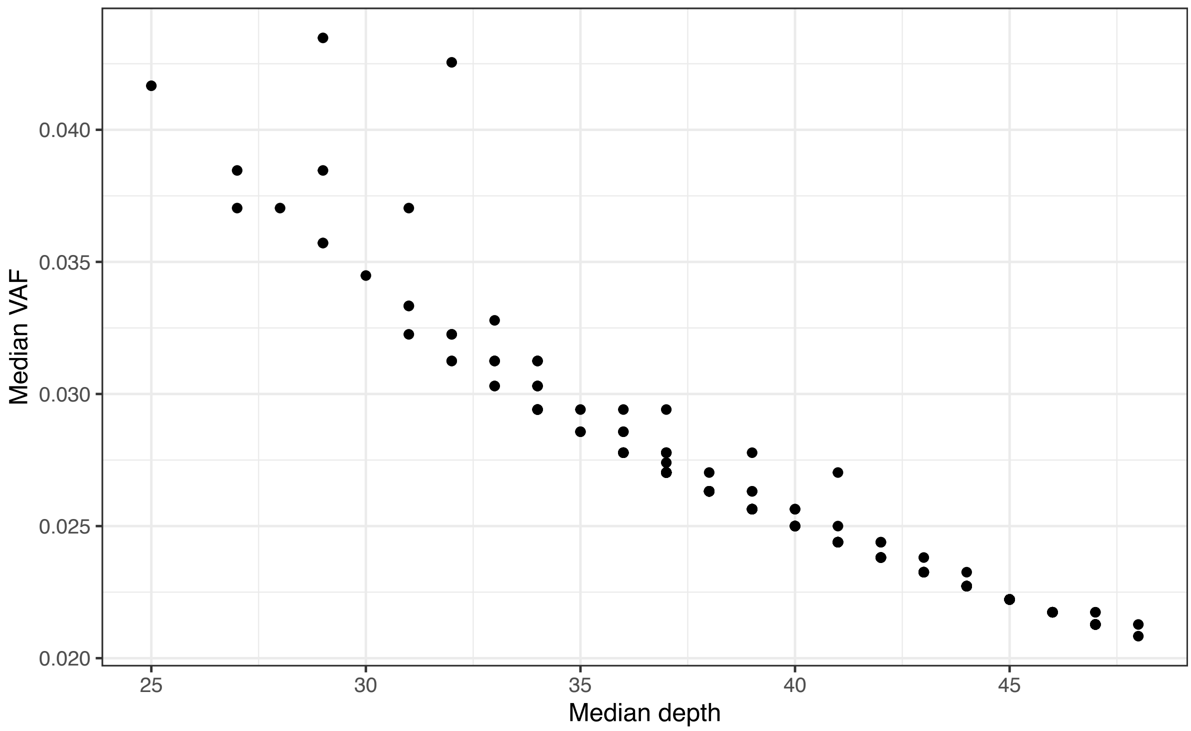

Finally, we can examine the number of variants at each position:

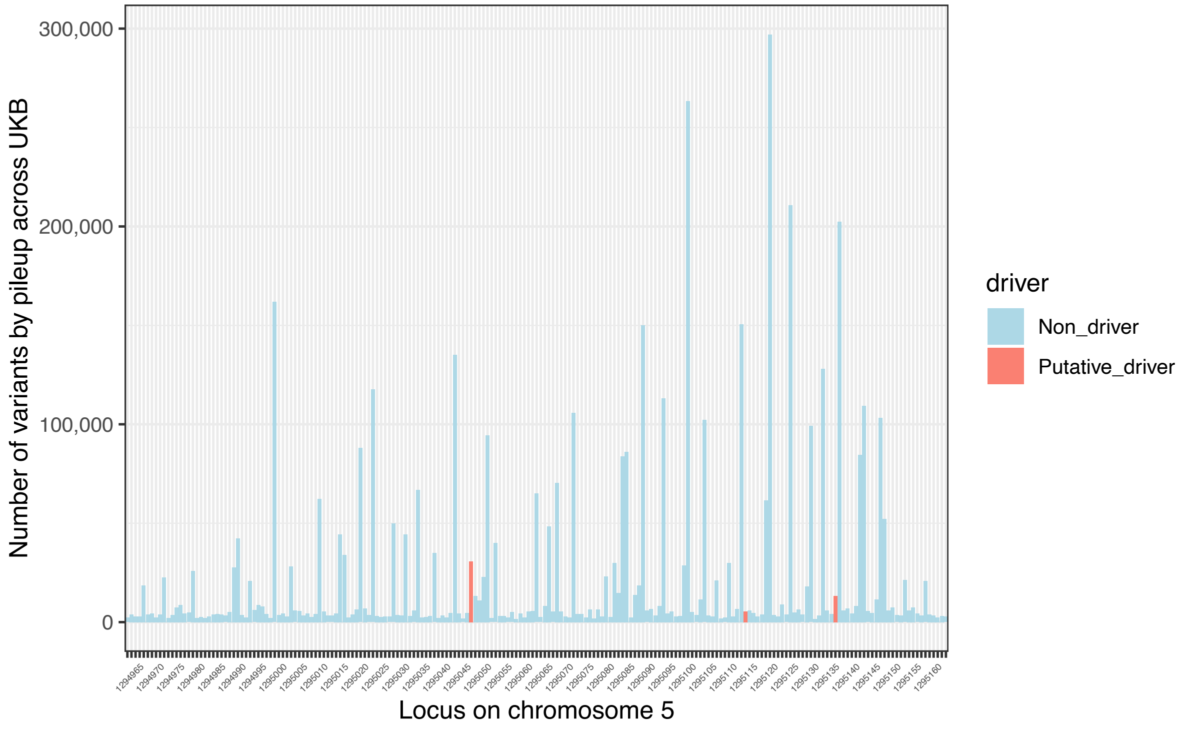

We can see that at some positions, there are huge numbers of alternate allele calls (that is, at least 1 alternate allele detected) - with one site having almost 300,000 variant calls across the UKB. Our three sites of interest seem to have a relatively low number of alternate allele calls, although one of the sites (chr5:1295046) has a higher number, suggesting this site might be more prone to error than the others.

We now start applying some relatively liberal arbitrary filters to the dataset to try and remove some of the noise and error and make the data more intelligible. We begin by applying a threshold of a minimum of 3 alternate reads, and also filtering sites with low depth (<15 reads) or a high VAF that might be

suggestive of germline contamination (VAF >30%). Finally, where an individual has more than one different alternate allele call at the same position, we consider only the variant with the highest number of alternate alleles (e.g. if an individual has reads for both C>T and C>A at position X with 5 and 4 reads respectively, we retain the C>T call and discard the C>A call). What does this do to the number of alternate allele calls across the UKB?

```
## [1] 105455     10
```

We have now reduced the number of alternate allele calls from 4.68 million to 105,455. This is a substantial reduction - but it is still much higher than might be expected for real somatic mutations, suggesting that erroneous calls remain in the dataset.

After applying these filters, how many variants are there at each position?

```
##
## 1294966 1294971 1294974 1294975 1294978 1294986 1294988 1294989 1294992
1294993
##       7       5       2       3       7       2      14      95       6
2
## 1294994 1294995 1294996 1294998 1295002 1295004 1295009 1295010 1295011
1295014
##       5       4       1    4878      14       1      77       1       1
100
## 1295015 1295018 1295019 1295020 1295022 1295023 1295024 1295027 1295030
1295033
##      33       1     656       2    1706       1       1      92      43
262
## 1295034 1295037 1295042 1295043 1295046 1295047 1295048 1295049 1295050
1295052
##      12      27    2486       1      60       1       4      15     813
66
## 1295053 1295055 1295056 1295060 1295062 1295063 1295065 1295067 1295068
1295071
##       3       1       1       1      71       5      64     179       5
1205
## 1295075 1295079 1295081 1295083 1295084 1295086 1295087 1295088 1295089
1295090
##       2       7      25     719     223       1       8     997       2
1
## 1295093 1295094 1295095 1295096 1295098 1295099 1295100 1295102 1295103
1295106
##     737       1       4       1      33   24563       2       2     485
11
## 1295107 1295109 1295111 1295112 1295113 1295114 1295118 1295119 1295121
1295122
##       4      15       2     736      80       1     242   38357       1
1
## 1295124 1295126 1295128 1295129 1295131 1295132 1295133 1295135 1295136
1295138
##   11681       4       5    1062       5    2274       1      13    6040
1
## 1295141 1295142 1295145 1295146 1295147 1295149 1295152 1295153 1295154
1295155
##     802    1716       1    1322     195       1      11       1       2
```

```
1
## 1295157 1295161
##      11       1
```

We can see that there are some sites with only a single, or sometimes a handful of alternate allele calls (which could be true passengers), others with tens of thousands, suggestive of error, and some with total numbers in between, representing a grey area where it is more difficult to classify variants as driver, passenger or error.

Specifically, after applying these initial filters, how many variants are there amongst our three putative drivers, and what is the distribution of the alternate allele calls (the base changes are driver-specific: chr5:1295046:T:G - NM_198253.3:c.-57A>C, chr5:1295113:G:A - NM_198253.3:c.-124C>T, and chr5:1295135:G:A - NM_198253.3:c.-146C>T).

```
##
##            A  G  T
##   1295046  0 60  0
##   1295113 79  0  1
##   1295135 12  0  1
```

At these sites, almost all the variants represent the expected driver mutation seen in telomere biology disorders. We can contrast this with the rest of the variants and try to visualize it with a stacked bar chart. Note: the y axis is on a log scale so that we can see the infrequently varying sites alongside the very frequently varying sites. This can be misleading as relatively modest differences in the proportions on log scale can reflect large differences on a linear scale.

Firstly, we examine the data before applying our filters (such as choosing the maximum at sites with more than one alternate allele call):

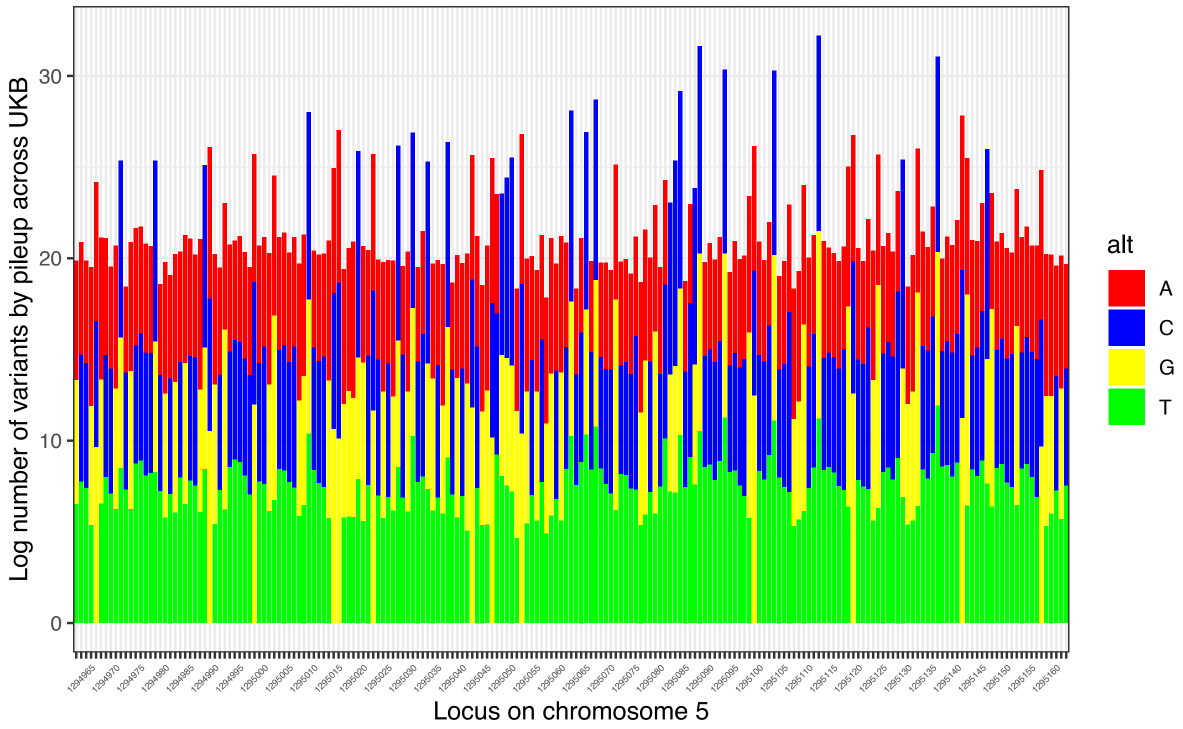

Next, we examine the data after filtering:

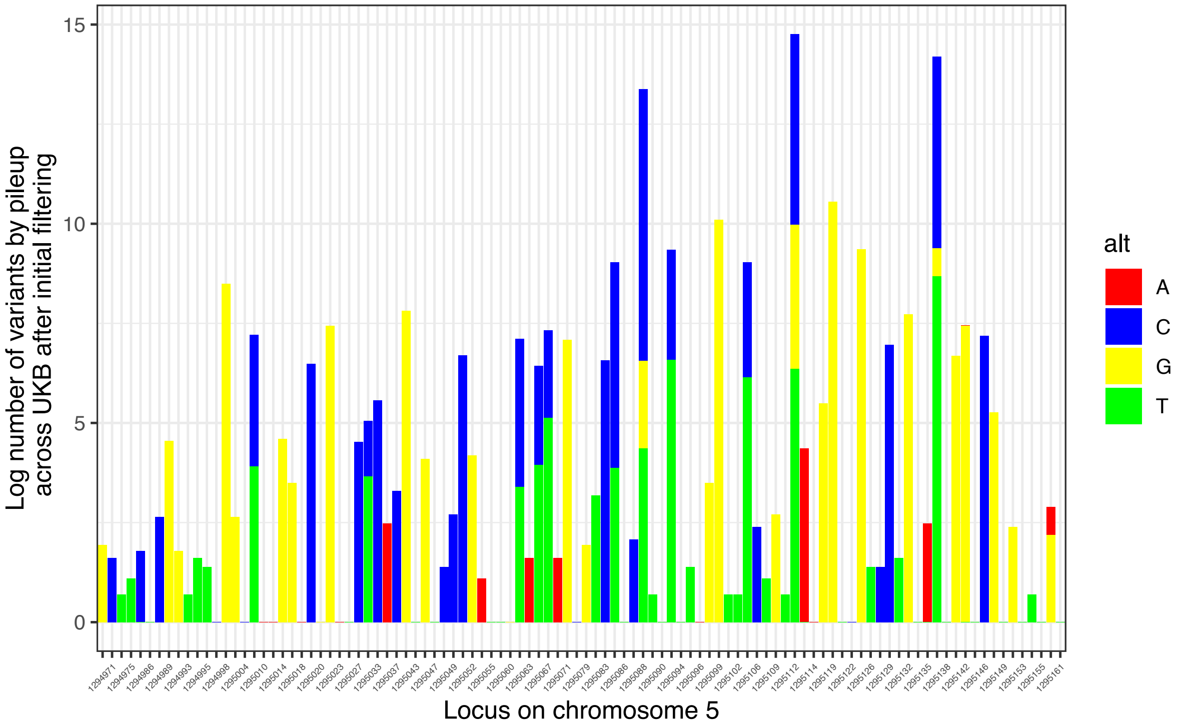

There are many sites where just a single alternate allele dominates after filtering by the minimum number of alternate reads and taking the alternative allele with the highest number of reads.

We now focus on our three sites of interest and the proportion of alternate allele calls before and after filtering. Because the numbers are more comparable here, we now use a linear scale on the y axis.

Before filtering:

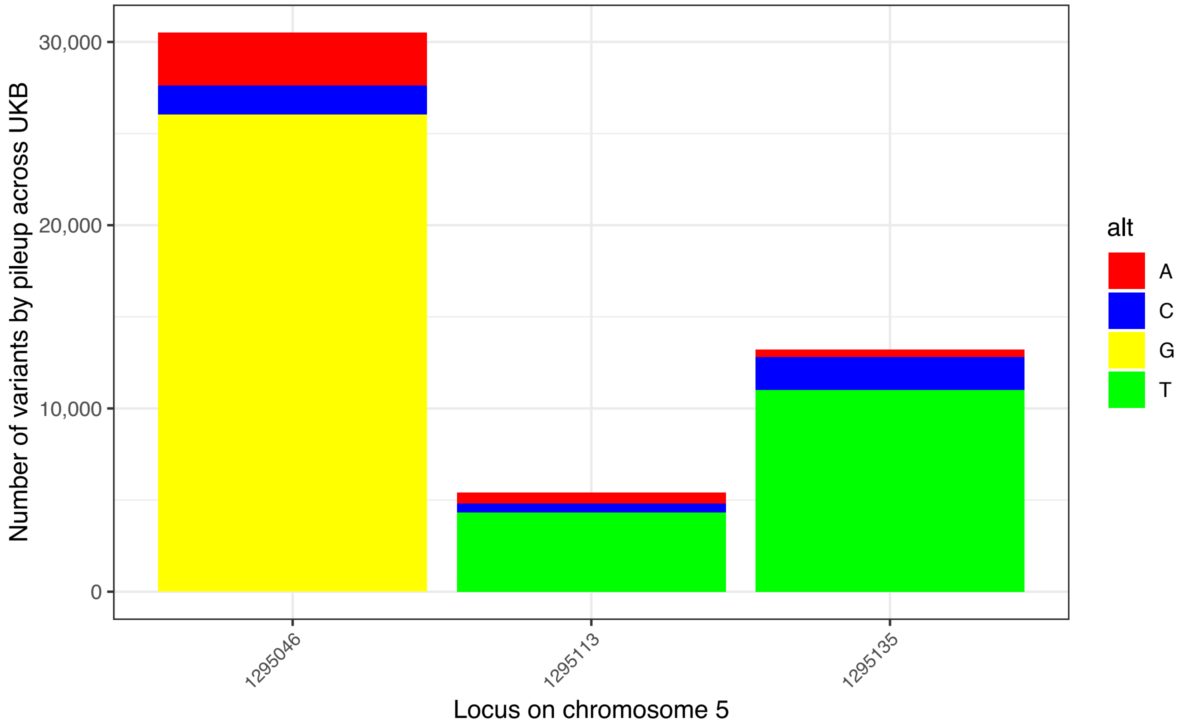

Note the very large number of calls before filtering.

After filtering, the number of calls is substantially reduced:

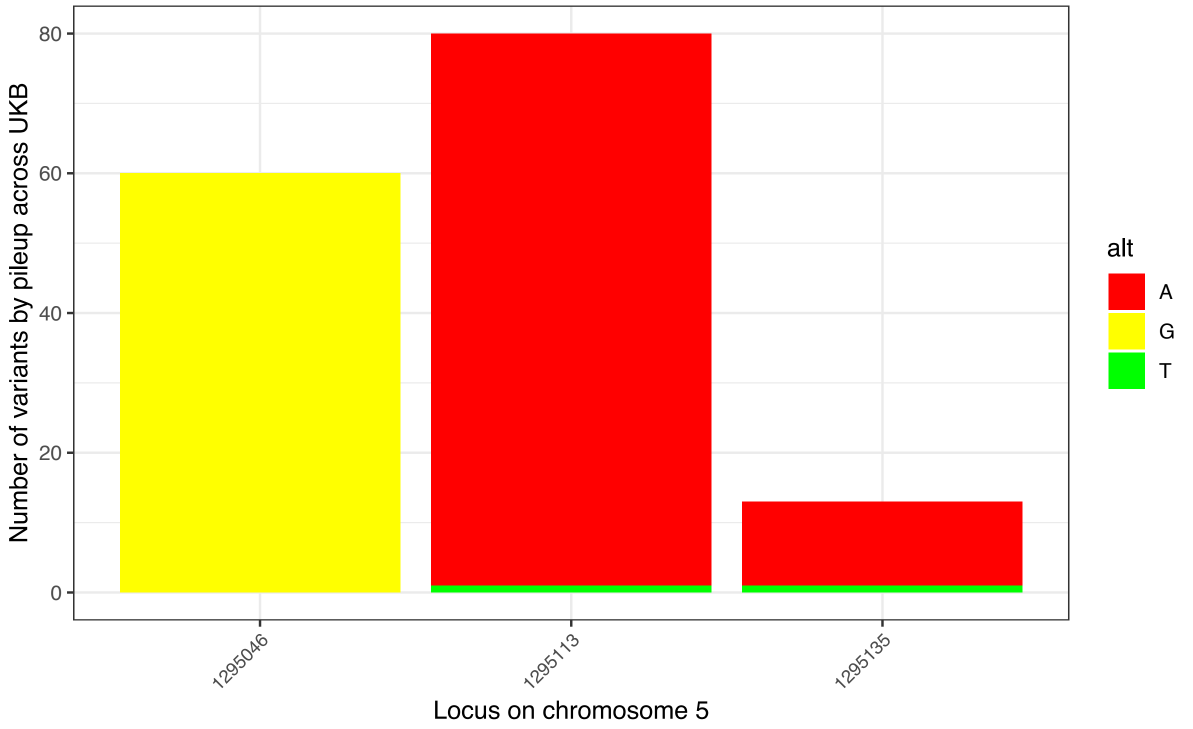

By applying our filters across the *TERT* promoter, we have enriched for what we believe to be “real” drivers at our sites of interest. It is worth noting again though, that at position chr5:1295046, unlike the other two positions, the “real” driver comprised the majority of alternate allele reads prior to filtering, so once again, we should pay special attention to this site, as it may be more likely than the other two positions to be contaminated by sequencing error.

Note that as a final filter at this stage, we have removed the two variants at our sites of interest that don’t have the expected alternate allele.

We will now move on to examining the distribution of the number and VAF of reads at each position in our filtered dataset. As we saw earlier, there are lots of sites with only a handful of alternate reads after filtering; since we cannot discern a “distribution” with so few data points, I have arbitrarily excluded those sites with <= 10 variants for this visualization step only. As there are a lot of data points, I have elected to make violin plots as visualizing individual data points makes for unintelligible graphs due to over-plotting. Here, we examine the distribution of the number of alternate reads at each site (note that at sites where all variants have 3 alternate reads, there is no distribution to plot):

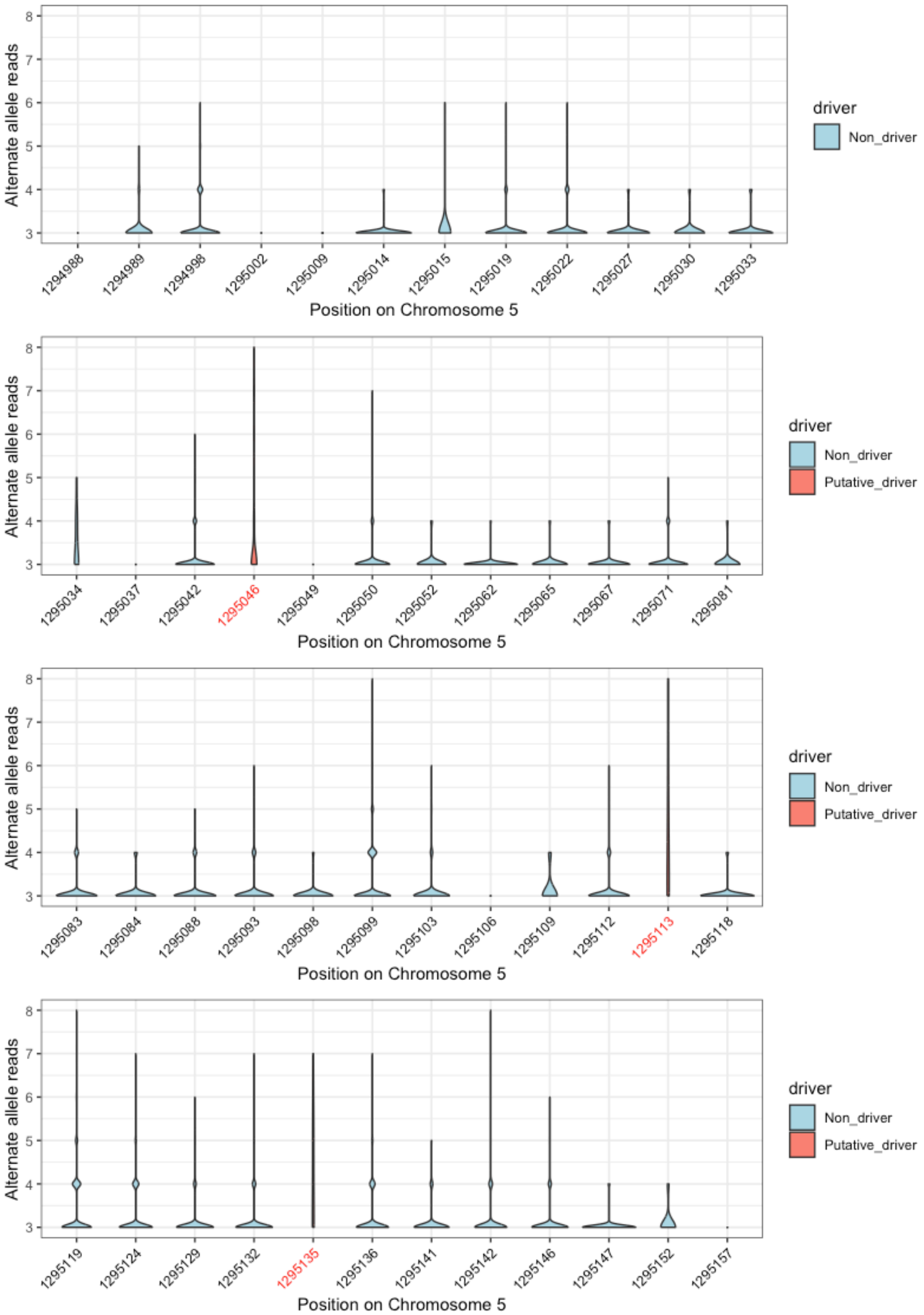

We can see that the distribution of the number of alternate reads is distinctly different at our three sites of interest, consistent with this being "real" and the other sites being largely sequencing noise. Of note, the distribution of the number of alternate reads at chr5:1295034 is very similar to our sites of interest, suggesting that this position might also be worth considering as a putative driver.

What are the alternate alleles at chr5:1295034?

```
##
##              A
##   1295034 12
```

They are all C>A mutations, and there are 12 individuals with these mutations.

We can also visualize the differences between distributions of alternate allele reads more formally by using the Mann-Whitney U test, computing n by n comparisons, then plotting the -log10 of the adjusted p values for these n by n comparisons in a heatmap. Since the computed p value is in part a function of sample size, we take a random sample of n=100 from sites where there are >100 variants passing filters (otherwise, at noisy sites with e.g. 10,000 variants passing our liberal filters, these have very large p values driven largely by their sample size). We see that our three sites of interest have the largest p values, in addition to the fourth site that we speculate may be an unreported driver:

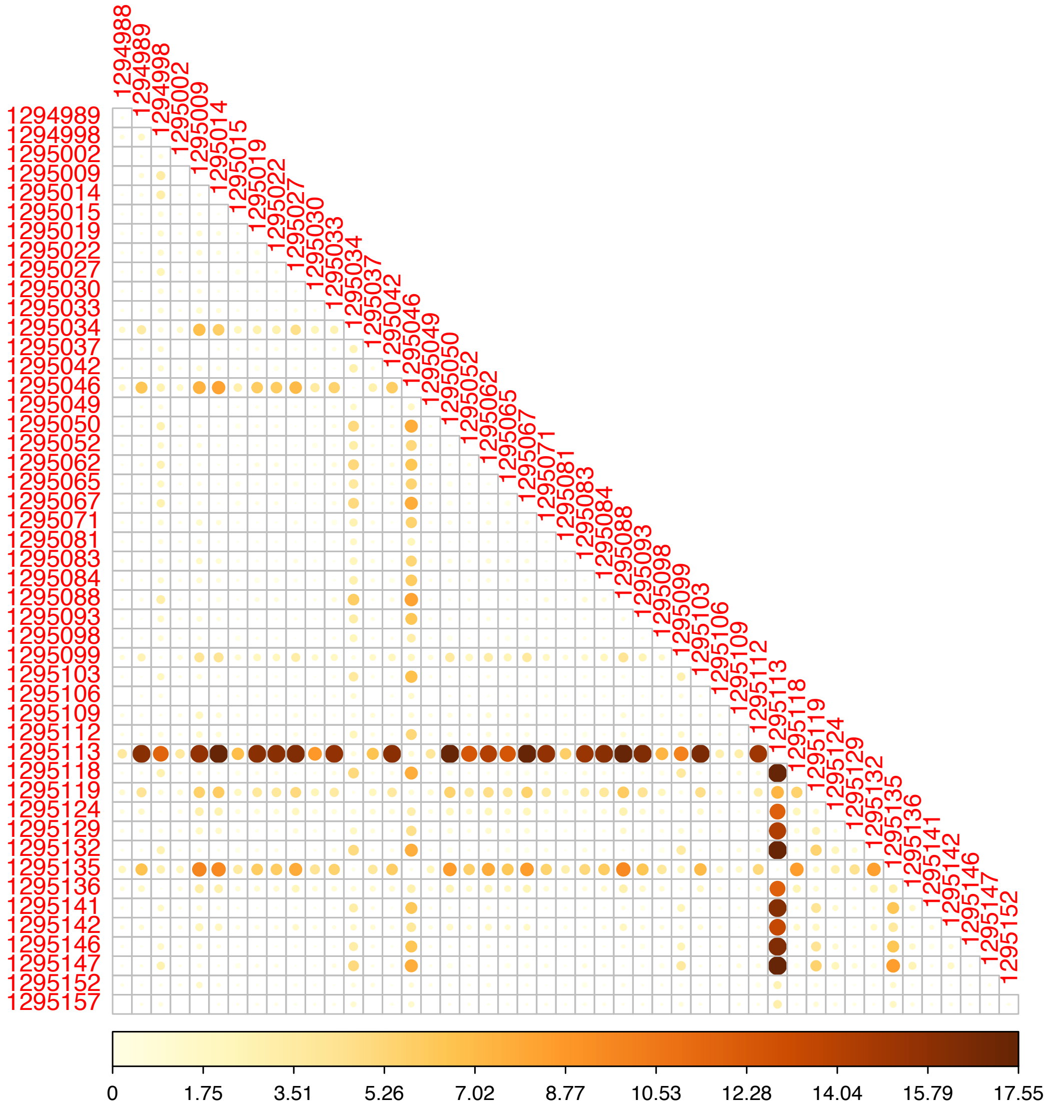

We can also visualize the distribution of VAFs across sites:

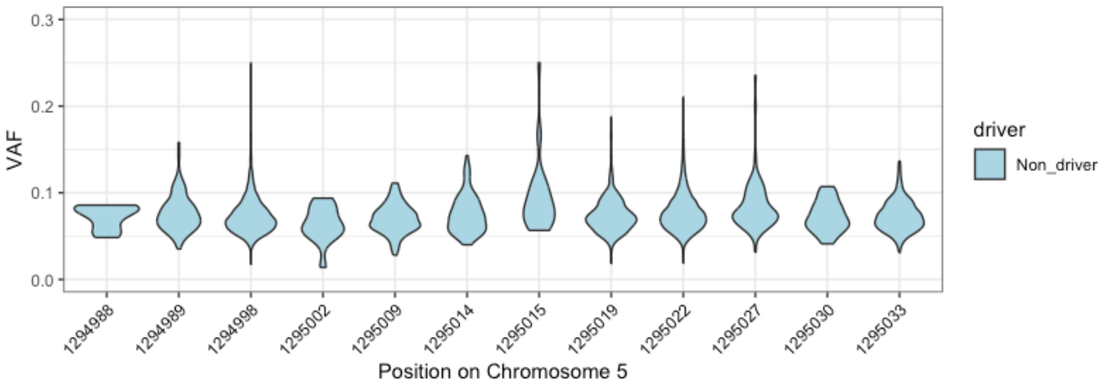

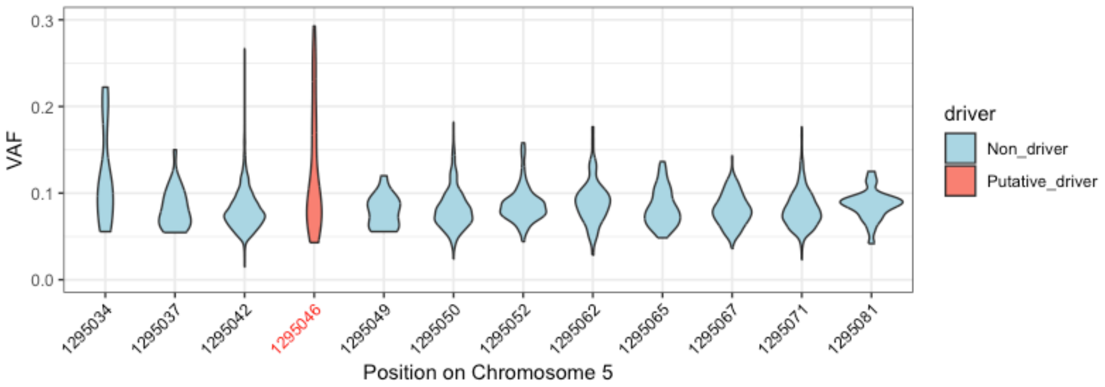

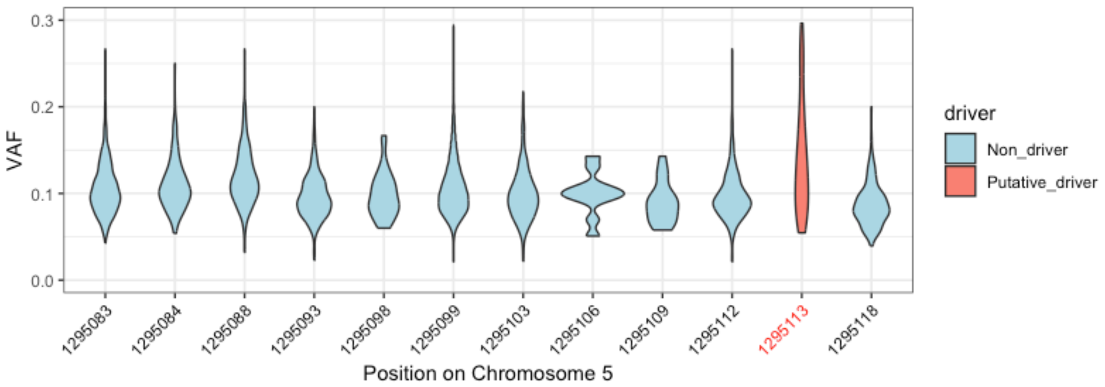

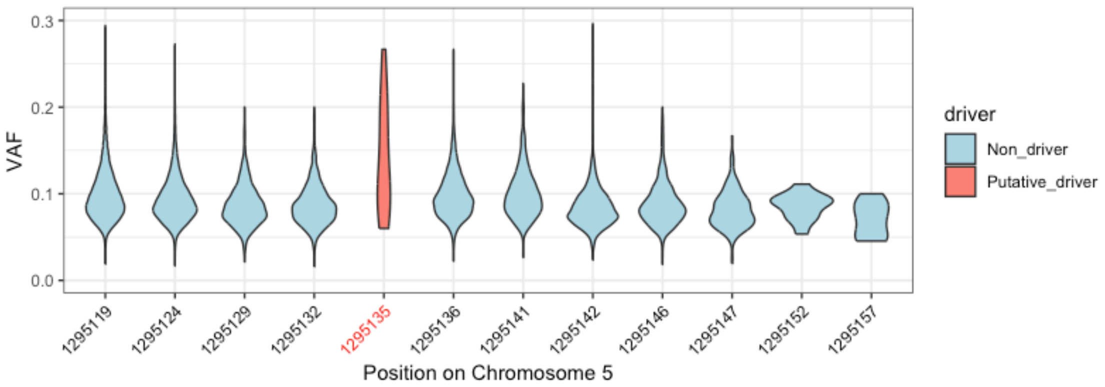

Again, we see that the distribution of VAFs are our site of interest looks distinct from the rest, and once again, the additional position of chr5:1295034 looks to have a distribution of VAFs that bears resemblance to our putative driver sites.

Association with polygenic risk score for leukocyte telomere length

When we visualize the distribution of polygenic risk scores at each position on the *TERT* promoter, our three sites of interest seem to have lower PRS. Of note, the previously discussed site chr5:1295034 once again shows a similar trend (though it is worth stressing there are only 12 individuals with mutations passing filters at this site):

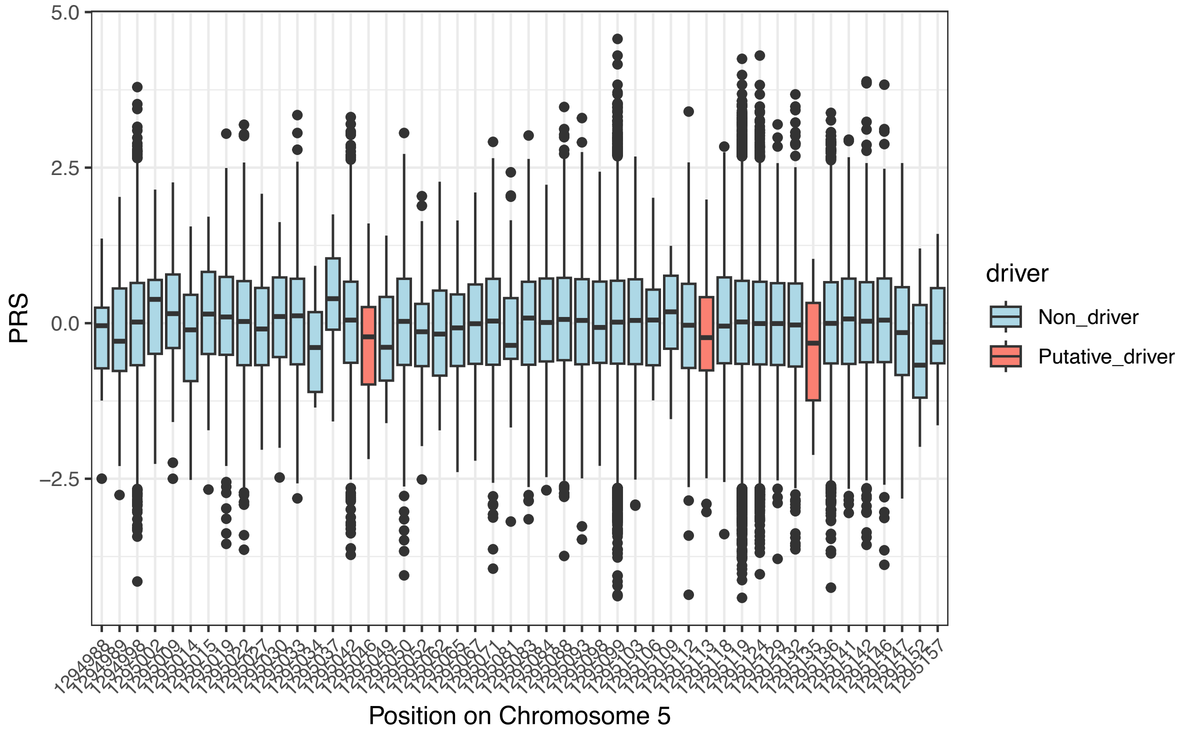

We can also make a pairwise comparison of the PRS of individuals with putative *TERT*p driver mutations with the rest of the UKB:

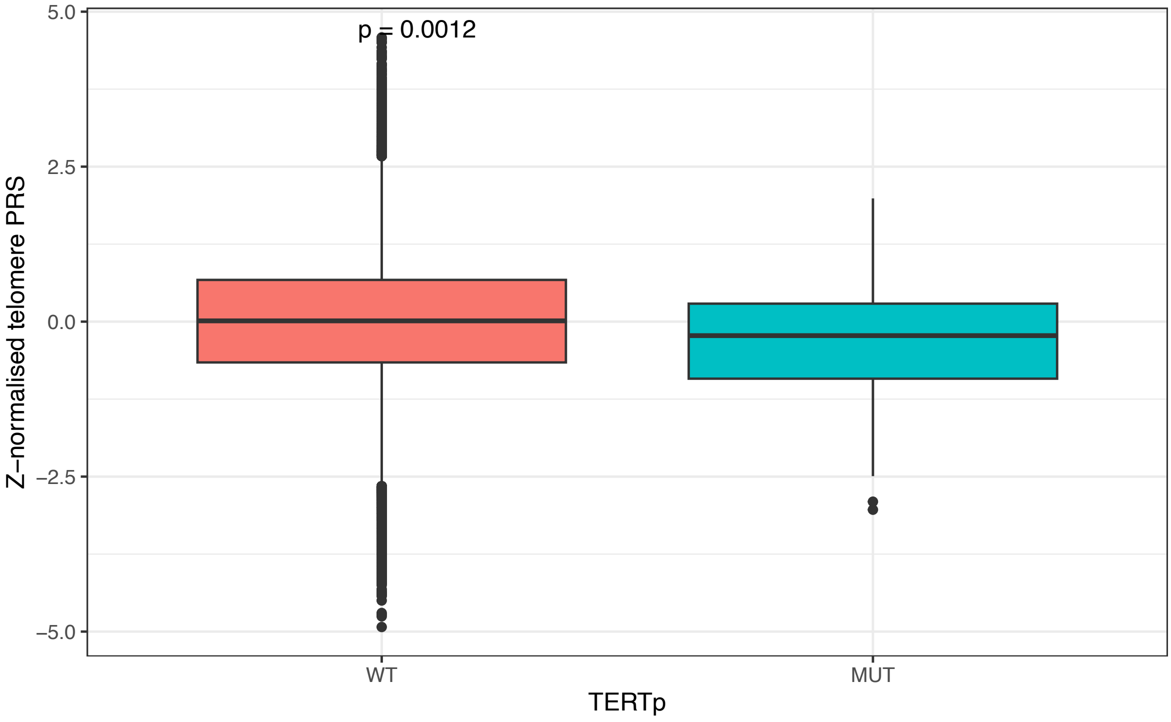

Age-related prevalence of TERTp putative drivers versus splicing mutations

We can also compare the prevalence of the *TERT*p driver mutations we have identified here with the prevalence of splicing factor mutations called from WGS data (using the thresholds and filters specified here), and contrast this with splicing factor, *DNMT3A* and *JAK2* mutations called from WES data using a more conventional Mutect2-based approach. Here, we are only comparing the combined prevalence of the *SF3B1* (R625, K666 and K700) and *SRSF2* (P95) hotspot mutations that we called using pileup, so that we are comparing like with like:

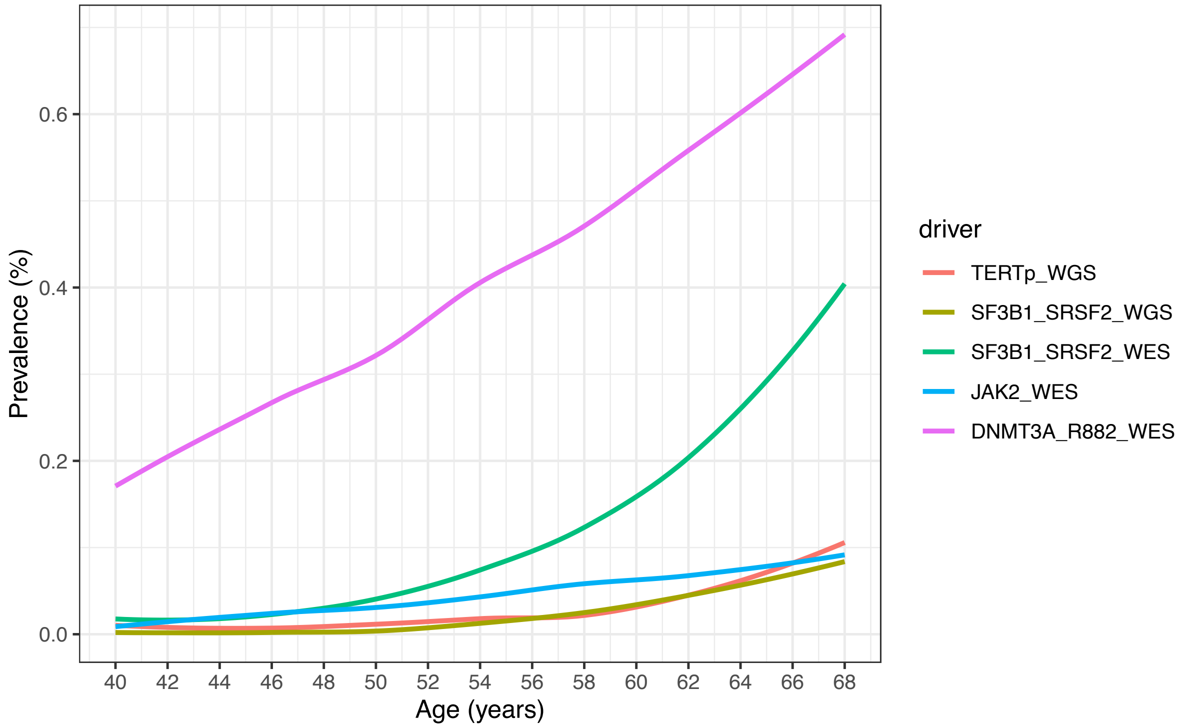

We can also reproduce the plot but distinguishing between *SRSF2* and *SF3B1*:

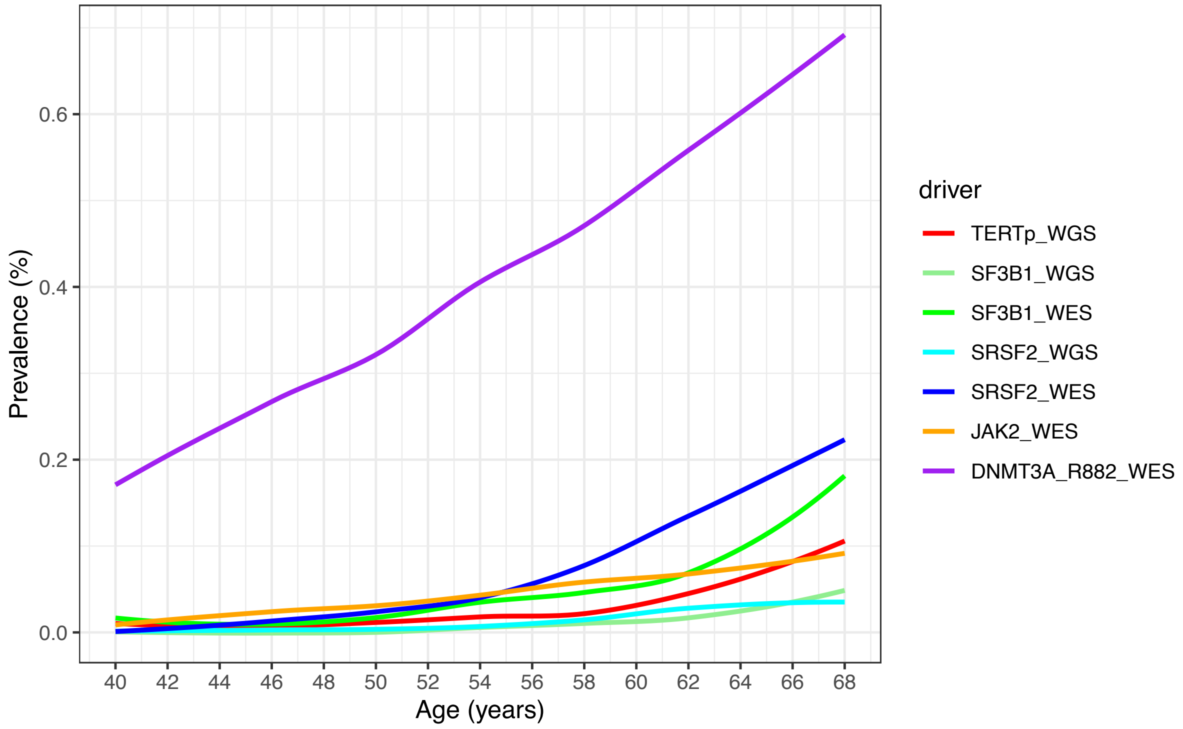

For clarity, we can also re-plot this removing the comparison with *DNMT3A* and *JAK2* hotspot mutation prevalence and colouring in different shades of green/blue for *SF3B1*/*SRSF2* respectively:

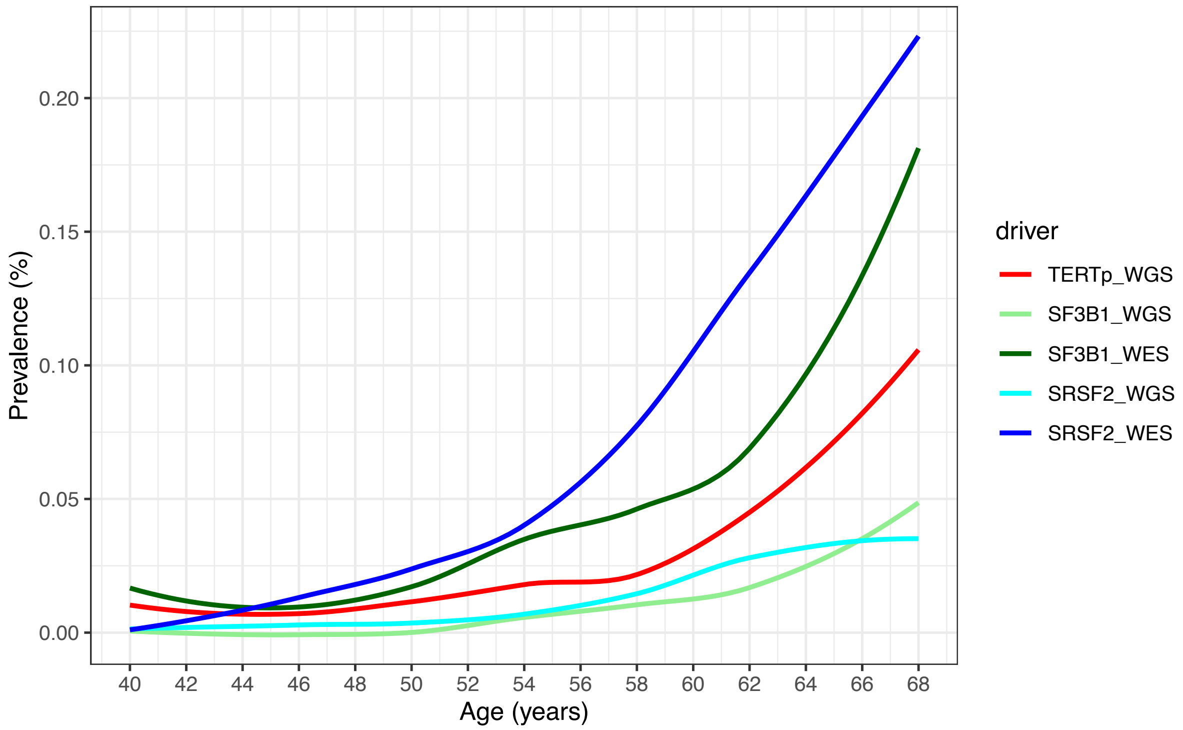

These plots show that, when we apply the same methodology to call splicing and *TERT*p hotspot mutations from WGS data, the prevalence of *TERT*p mutations appears similar to the combined prevalence of CH driven by *SF3B1* and *SRSF2* mutations.

To benchmark our ability to call hotspot mutations from WGS using the liberal filtering method described here, we can compare the overlap between *SF3B1*/*SRSF2* called using our described filtering of WGS pileup, versus calling this using Mutect2 on WES (note here that we are using the subset of UKB participants who have both WES and WGS, as ~490k have WGS c.f. only ~450k have WES):

Overlap of SF3B1 calls between WGS (pileup) and WES (Mutect2)

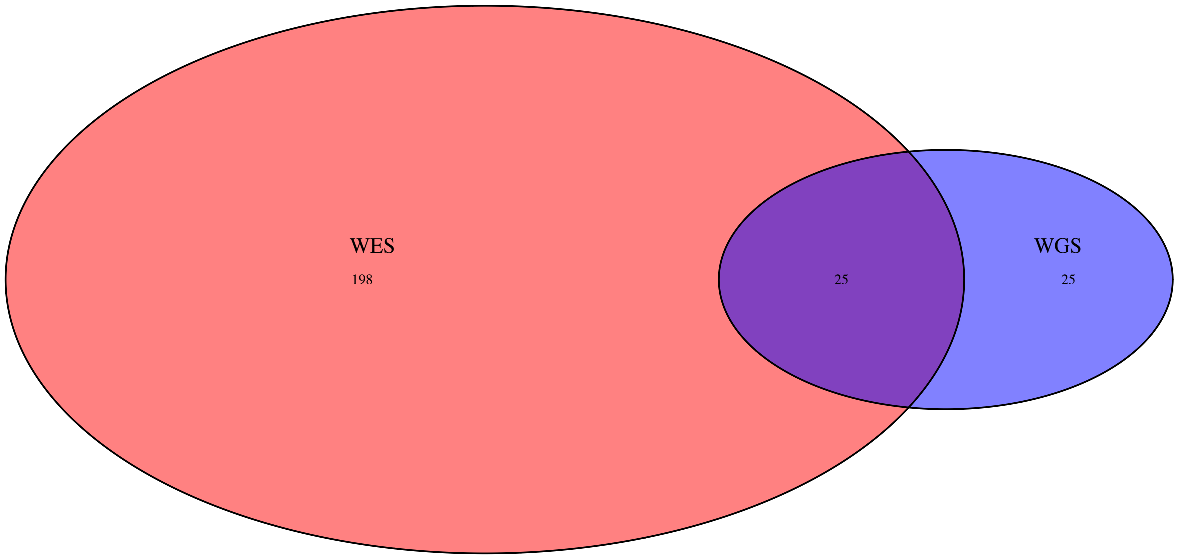

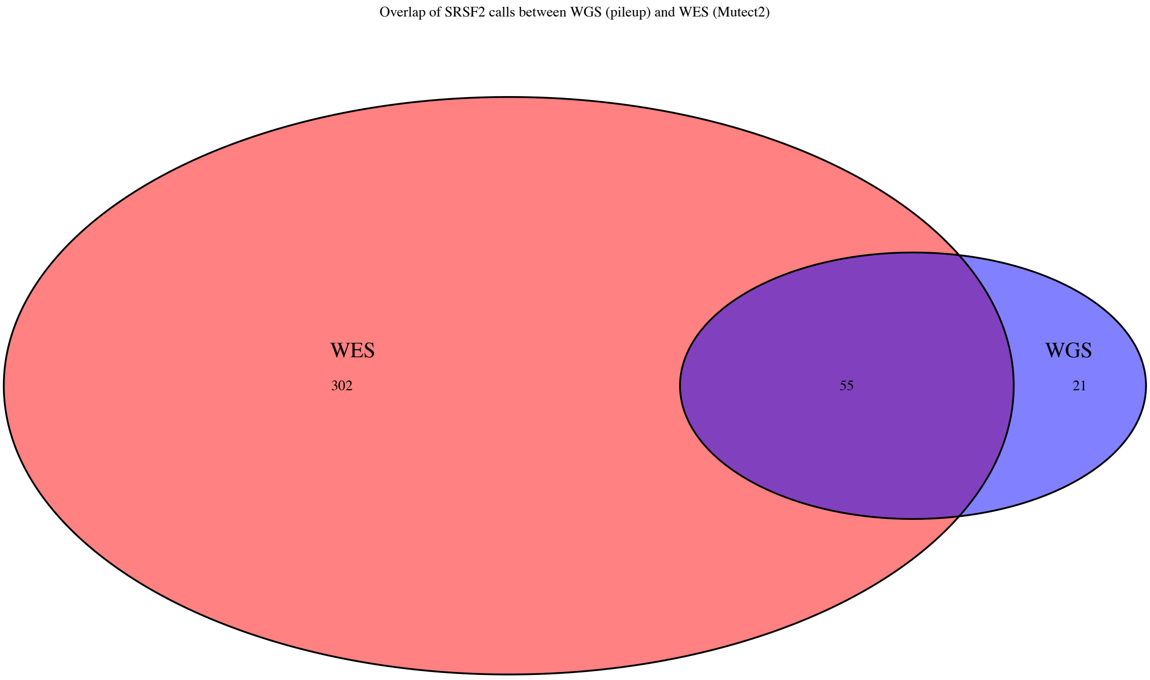

We see that there are a number of mutations that are only detected by pileup on WGS, despite the lower depth of WGS. To examine whether these appear to be "real" calls (versus error), we next examine whether these WGS-exclusive calls associate with macrocytosis (*SF3B1*) and thrombocytopenia (*SRSF2*) (that is, do they exhibit known blood count phenotypes associated with these mutations). The reference (leftmost) group in both cases is a cohort of age and sex matched controls where the sample size is ten-fold greater than the number of *SF3B1*/*SRSF2* cases respectively.

We can see that our pileup calls for *SF3B1* have a higher MCV than an age- and sex-matched cohort:

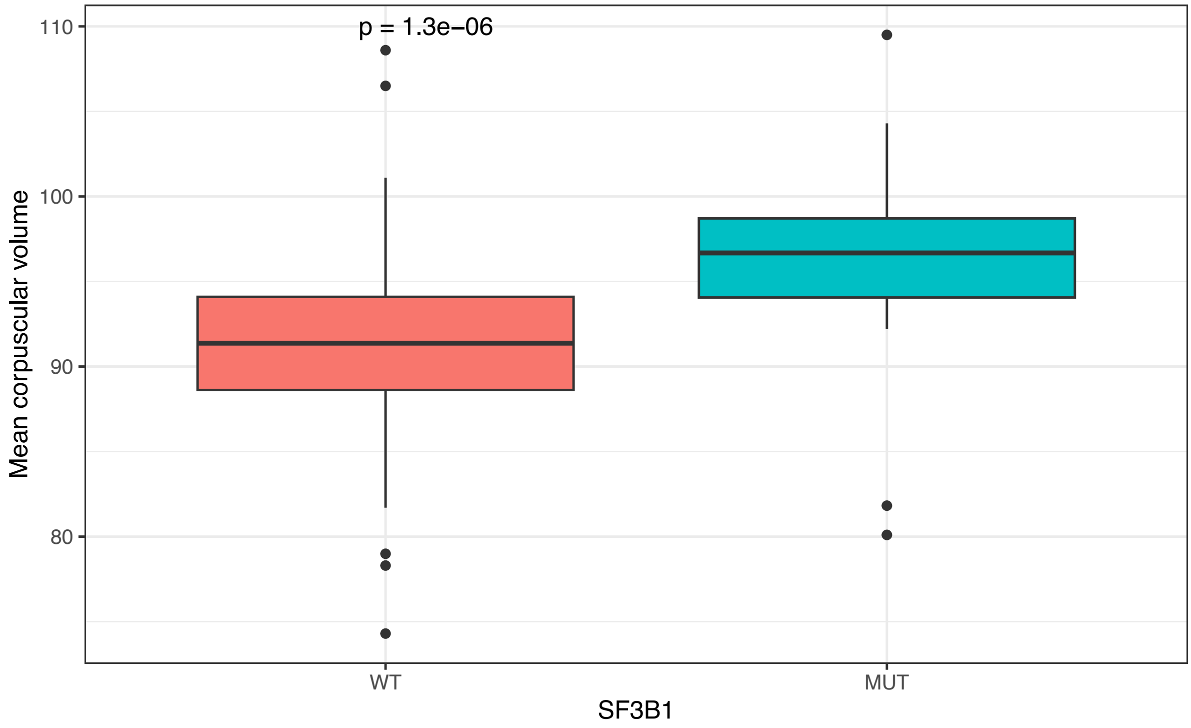

And our *SRSF2* calls by pileup have lower platelet counts:

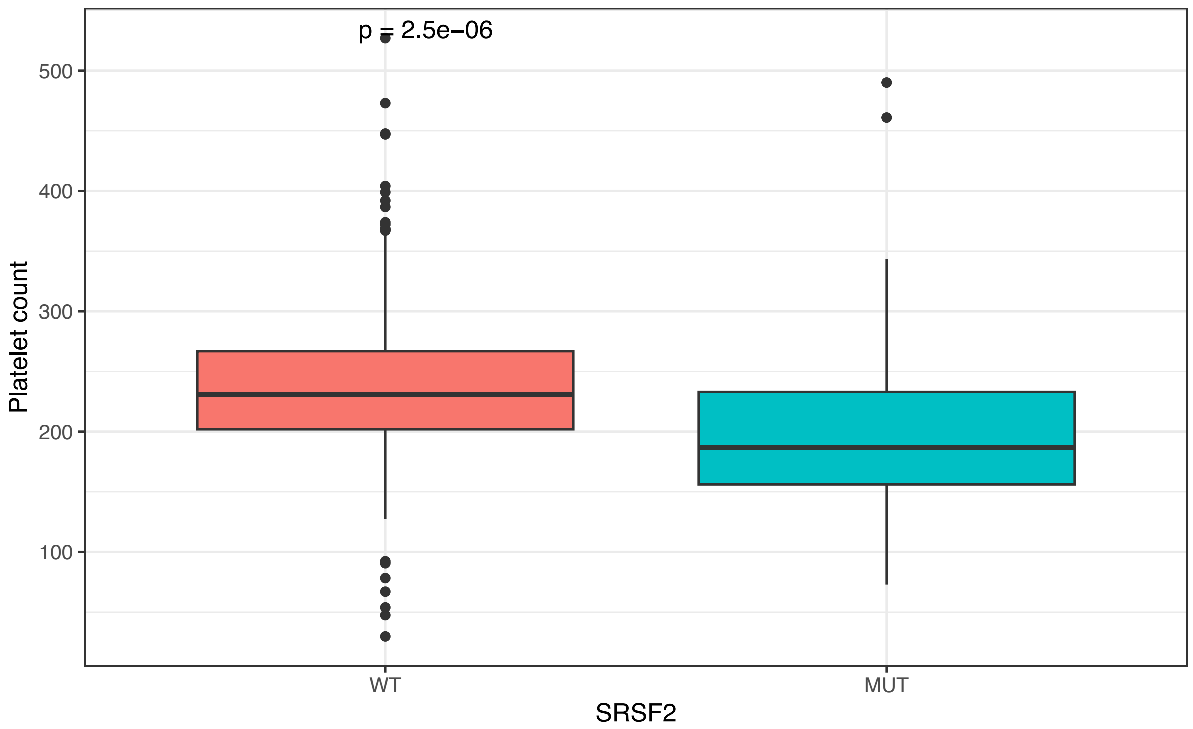

Association between TERTp variants and lymphocyte count

We wanted to ensure that individuals with *TERT*p mutations did not have a higher relative lymphocyte count (relative to granulocytes), since this might suggest that these mutations were present in a lymphoid progenitor, rather than an HSC or myeloid progenitor.

Firstly, we plot the absolute lymphocyte count vs position on the *TERT* promoter, to check whether or not these individuals have a phenotype suggestive of unannotated CLL or MBL:

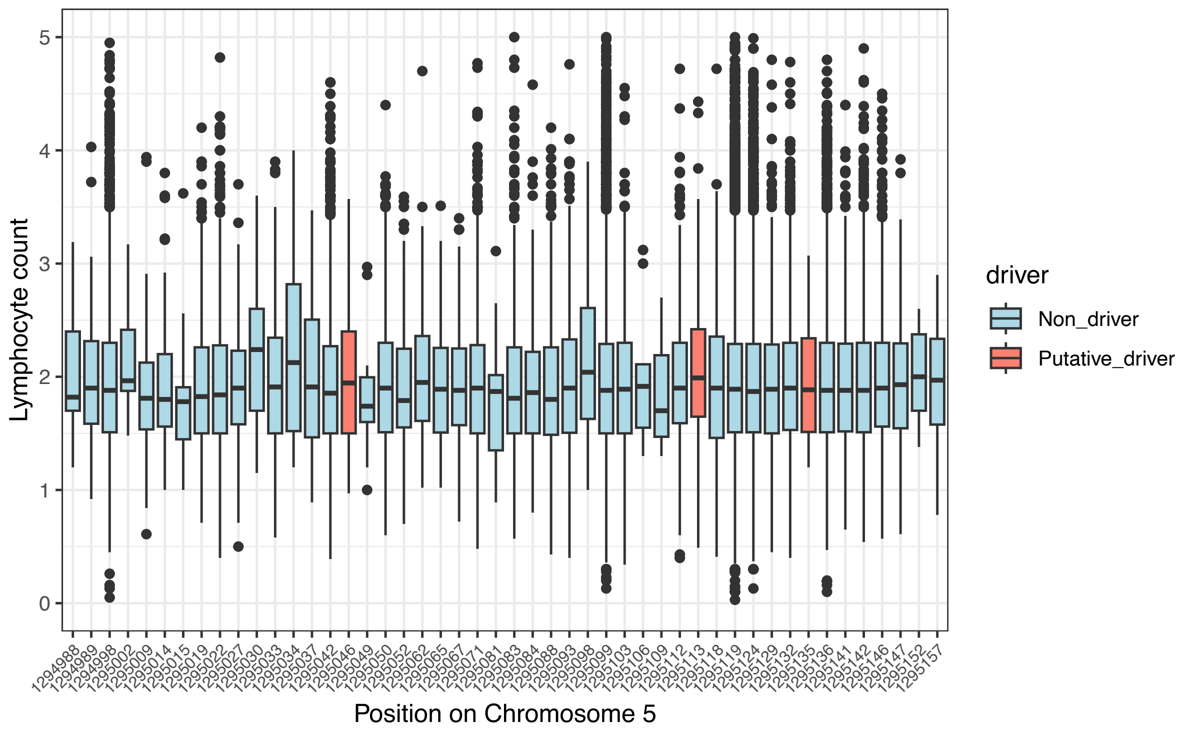

Here, we don't see any strong association between the absolute lymphocyte count and the presence of these putative *TERT*p driver mutations.

We can also look at the VAF and color by the lymphocyte percentage to examine whether those with a high VAF have a high lymphocyte percentage (consistent with the TERTp mutations being present in a lymphocyte progenitor):

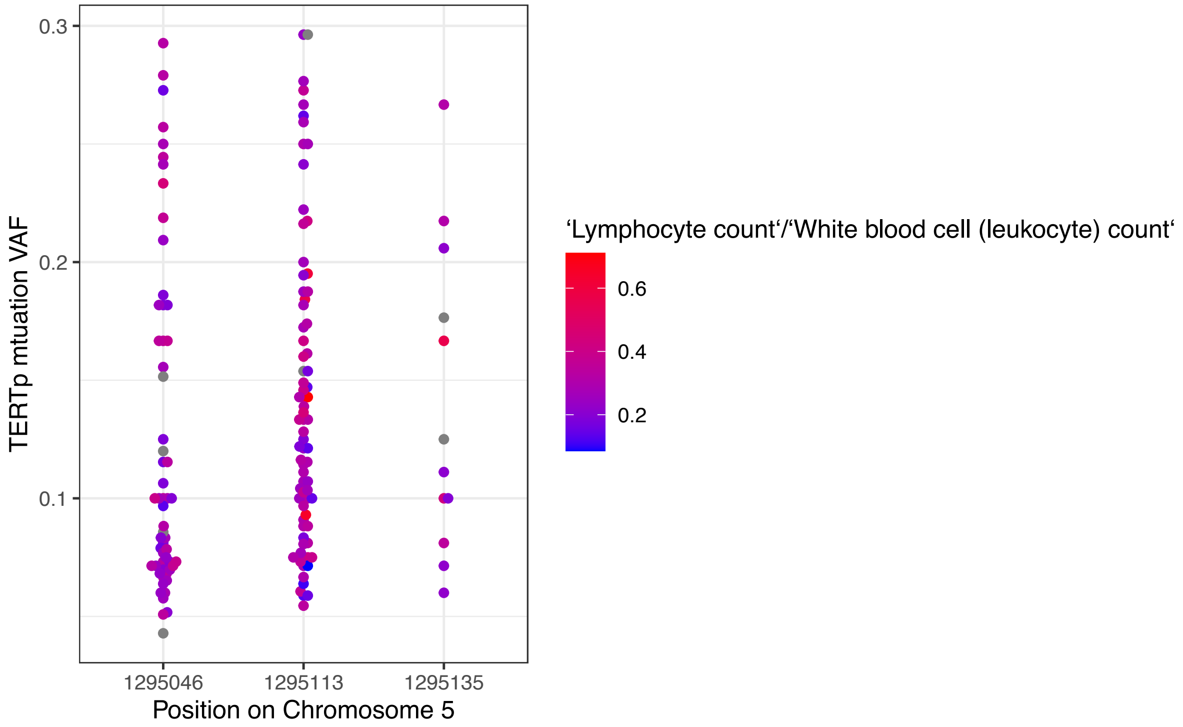

Alternatively, we can directly plot VAF vs lymphocyte percentage across the three sites:

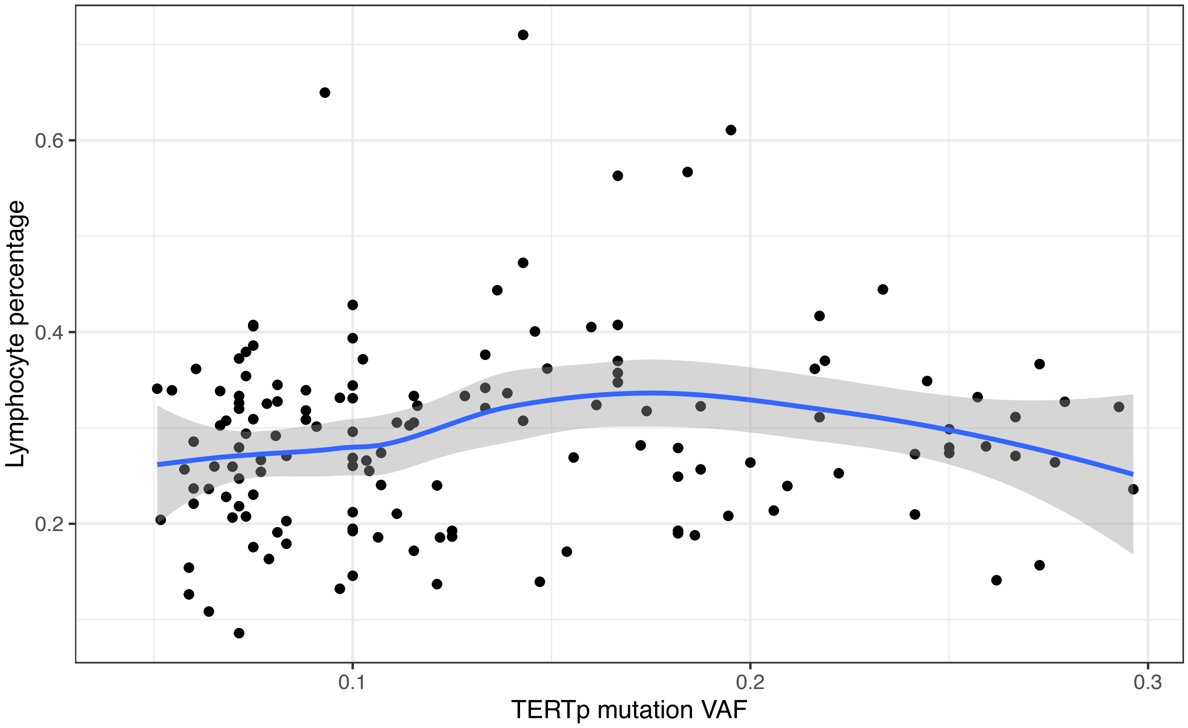

We can see that there is no apparent association between TERTp mutation VAF and lymphocyte percentage, and therefore no evidence to suggest that these mutations are present in a lymphoid population.

Supplementary References

1. Gutierrez-Rodrigues, F. *et al.* Clonal landscape and clinical outcomes of telomere biology disorders: somatic rescuing and cancer mutations. *Blood* blood.2024025023 (2024) doi:10.1182/blood.2024025023.
2. Cawthon, R. M. Telomere measurement by quantitative PCR. *Nucleic Acids Research* **30**, 47e–447 (2002).
3. McKerrell, T. *et al.* Leukemia-Associated Somatic Mutations Drive Distinct Patterns of Age-Related Clonal Hemopoiesis. *Cell Reports* **10**, 1239–1245 (2015).
4. Park, N. & Vassiliou, G. Design and Application of Multiplex PCR Seq for the Detection of Somatic Mutations Associated with Myeloid Malignancies. in *Acute Myeloid Leukemia* (eds. Fortina, P., Londin, E., Park, J. Y. & Kricka, L. J.) vol. 1633 87–99 (Springer New York, New York, NY, 2017).
5. Li, H. & Durbin, R. Fast and accurate short read alignment with Burrows–Wheeler transform. *Bioinformatics* **25**, 1754–1760 (2009).
6. Danecek, P. *et al.* Twelve years of SAMtools and BCFtools. *Gigascience* **10**, giab008 (2021).
