# Supplementary material for: Telomere attrition becomes an instrument for clonal selection in aging hematopoiesis and leukemogenesis
Source: Nat Genet. 2025 Aug 28;57(9):2215–25. doi: 10.1038/s41588-025-02296-x (PMC12425810; doi:10.1038/s41588-025-02296-x)
Supplement: Supplementary file 2 — Reporting Summary [file 41588_2025_2296_MOESM2_ESM.pdf]

nature portfolio

Corresponding author(s): George Vassiliou

Last updated by author(s): Jun 2, 2025

Reporting Summary

Nature Portfolio wishes to improve the reproducibility of the work that we publish. This form provides structure for consistency and transparency in reporting. For further information on Nature Portfolio policies, see our Editorial Policies and the Editorial Policy Checklist.

Statistics

For all statistical analyses, confirm that the following items are present in the figure legend, table legend, main text, or Methods section.

n/a	Confirmed	
☐	☒	The exact sample size (*n*) for each experimental group/condition, given as a discrete number and unit of measurement
☐	☒	A statement on whether measurements were taken from distinct samples or whether the same sample was measured repeatedly
☐	☒	The statistical test(s) used AND whether they are one- or two-sided<br>*Only common tests should be described solely by name; describe more complex techniques in the Methods section.*
☐	☒	A description of all covariates tested
☐	☒	A description of any assumptions or corrections, such as tests of normality and adjustment for multiple comparisons
☐	☒	A full description of the statistical parameters including central tendency (e.g. means) or other basic estimates (e.g. regression coefficient) AND variation (e.g. standard deviation) or associated estimates of uncertainty (e.g. confidence intervals)
☐	☒	For null hypothesis testing, the test statistic (e.g. *F*, *t*, *r*) with confidence intervals, effect sizes, degrees of freedom and *P* value noted<br>*Give P values as exact values whenever suitable.*
☒	☐	For Bayesian analysis, information on the choice of priors and Markov chain Monte Carlo settings
☒	☐	For hierarchical and complex designs, identification of the appropriate level for tests and full reporting of outcomes
☒	☐	Estimates of effect sizes (e.g. Cohen's *d*, Pearson's *r*), indicating how they were calculated

Our web collection on statistics for biologists contains articles on many of the points above.

Software and code

Policy information about availability of computer code

Data collection	Data collected by the UK Biobank was either downloaded or accessed through the UK Biobank research analysis platform. Data collected by the All of Us study can be accessed at https://researchallofus.org/. Data used to generate phylogenetic trees was collected from individuals with informed consent obtained from either the Cambridge Blood and Stem Cell Biobank (REC 18/EE/0199) or from the SardiNIA longitudinal study, who had single-HSPC derived colonies collected as part of a previously approved study into immunosenescence (REC 15/EE/0327).
Data analysis	We used Mutect2 GATK v. 4.1.3.0 to identify somatic variants in the CH driver genes. All UK Biobank associated linear and logistic regression analyses were performed using the Python (v. 3.9.7) module statmodels (v. 0.12.2). Polygenic risk scores were calculated with the help of PRSice-2 (v. 2.3.5). Association between genetic variants and CH subtypes were determined by Firth's logistic regression analysis performed using the logistf function in R (v. 4.2.1) TwoSampleMR package (v. 0.5.7) in R (v. 4.3.0) was used to perform the Mendelian Randomization analysis. For phylogenetic analyses, all code used to generate phylogenies from single-HSPC derived colonies is available on GitHub: single nucleotide variants (SNVs) were called using the cancer variants through expectation maximization (CaVEMan) algorithm (https://github.com/cancerit/CaVEMan), indels were called using Pindel (https://github.com/cancerit/cgpPindel), whilst allele counts at SNV/indel sites were calculated using vafCorrect (https://github.com/cancerit/vafCorrect). Rtreemut and rtreefit, used to assign mutations to branches and infer temporal branch lengths/mutation rates respectively, are available from https://github.com/nangalialab/treemut and https://github.com/nangalialab/rtreefit. Telomerecat, used to estimate colony telomere lengths from WGS, can be accessed from: (https://github.com/telomerecat). The pairwise comparisons of splicing mutant clades and construction of linear mixed-effects models were carried out using custom R scripts available at: https://github.com/billydunn/telomeres-ch. Python code used to perform the primary LTL-CH and PRS-CH association analyses in the UKB and the R script for MR analysis are available at https://github.com/cksruthi/telomeres-CH. Scripts used for the analysis of targeted sequencing data can also be found at https://github.com/cksruthi/telomeres-CH.

For manuscripts utilizing custom algorithms or software that are central to the research but not yet described in published literature, software must be made available to editors and reviewers. We strongly encourage code deposition in a community repository (e.g. GitHub). See the Nature Portfolio guidelines for submitting code & software for further information.

Data

Policy information about availability of data

All manuscripts must include a data availability statement. This statement should provide the following information, where applicable:

- Accession codes, unique identifiers, or web links for publicly available datasets
- A description of any restrictions on data availability
- For clinical datasets or third party data, please ensure that the statement adheres to our policy

UKB data is publicly available, and access can be requested at https://www.ukbiobank.ac.uk. All of Us data can be accessed at https://researchallofus.org/. Targeted amplicon sequencing data has been uploaded to SRA (PRJNA1121075). Other data generated or analyzed in this study are available on reasonable request.

Research involving human participants, their data, or biological material

Policy information about studies with human participants or human data. See also policy information about sex, gender (identity/presentation), and sexual orientation and race, ethnicity and racism.

Reporting on sex and gender	For UK Biobank based analyses participants with matching self-reported and genetic sex were included. 54% of the participants were women. Sex was used as a covariate in all association analyses. For clinical samples, sex- or gender-based analysis was not performed due to the small sample size used.
Reporting on race, ethnicity, or other socially relevant groupings	First ten principal components (PCs) of genetic ethnicity were used as covariates in several of the UK Biobank analyses. Details on how the PCs were determined can be found at Bycroft, Clare, et al. BioRxiv (2017): 166298. For clinical samples, information on race, ethinicity and other socially relevant groupings were not available.
Population characteristics	For UK Biobank based analyses we used data of n=454,098 participants (age range:, 37-73, 54% female) for whom whole exome sequencing data was available as of November 2021. For clinical samples, patient characteristics are provided in the Supplementary Tables.
Recruitment	For clinical samples, recruitment was carried out by the Manchester Cancer Research Centre (MCRC) Biobank or the Cambridge Blood and Stem Cell Biobank (CBSB) and involved obtaining informed consent from patients undergoing investigation or treatment of a suspected/confirmed hematological condition. Details of participant recruitment for the UK Biobank can be found at https:/www.ukbiobank.ac.uk and in Sudlow C, et al. (2015) PLoS Med 12(3):e1001779. This study involves data of 454,098 (age range:, 37-73, 54% females) individuals from the UK Biobank with whole exome sequencing data released as of November 2021.
Ethics oversight	Clinical samples were obtained with informed consent from the Cambridge Blood and Stem Cell Biobank (CBSB) with approval from the Cambridge East Research Ethics Committee (REC: 18/EE/0199 and 24/EE/0116), from individuals in the SardiNIA longitudinal study of immunosenescence (REC 15/EE/0327) or from the Manchester Cancer Research Centre (MCRC) Biobank with approval of the South Manchester Research Ethics Committee (REC 07/H1003/161+5; HTA license 30004). The UK Biobank resource was approved by the North West Multi-centre Research Ethics Committee under reference number 21/NW/0157 and all participants provided written, informed consent to participate. Participants in the UK Biobank are volunteers and did not receive compensation for their involvement. Data for this study were accessed under approved application number 56844 from the UK Biobank resource.

Note that full information on the approval of the study protocol must also be provided in the manuscript.

Field-specific reporting

Please select the one below that is the best fit for your research. If you are not sure, read the appropriate sections before making your selection.

☒ Life sciences ☐ Behavioural & social sciences ☐ Ecological, evolutionary & environmental sciences

For a reference copy of the document with all sections, see nature.com/documents/nr-reporting-summary-flat.pdf

Life sciences study design

All studies must disclose on these points even when the disclosure is negative.

Sample size	For UK Biobank based analyses we used data of n=454,098 participants (age range:, 37-73, 54% females for whom whole exome sequencing data was available as of November 2021. For the analyses of clinical samples in Figures 3 and 4, we included all available samples which met our criteria.
Data exclusions	Individuals with no leukocyte telomere length measurements and imputed genotypic data were excluded. Additionally, participants who had withdrawn consent, or had a mismatch between genetic and self-reported sex, or had differences in the dates of attending the assessment center and the blood sample collection, were excluded from the study.
Replication	Replication was performed by applying the UK Biobank-derived leukocyte telomere length polygenic risk score (LTL-PRS) score to the All of Us cohort, and by applying the TOPMed-derived LTL-PRS score to the UK Biobank cohort.

Randomization	The experiments were not randomized. Age at recruitment, sex, smoking status, first ten genetic principal components were included as covariates in the association analysis with polygenic risk score and telomere length. WBC counts and percentages of WBC types were also included as covariates in analyses involving telomere length.
Blinding	The experiments were conducted without blinding due to feasibility. In UK Biobank analyses, CH mutations vary substantially in prevalence, such that the number of individuals with a given CH mutation can reveal the nature of the mutation and make blinding ineffective.

Behavioural & social sciences study design

All studies must disclose on these points even when the disclosure is negative.

Study description	*Briefly describe the study type including whether data are quantitative, qualitative, or mixed-methods (e.g. qualitative cross-sectional, quantitative experimental, mixed-methods case study).*
Research sample	*State the research sample (e.g. Harvard university undergraduates, villagers in rural India) and provide relevant demographic information (e.g. age, sex) and indicate whether the sample is representative. Provide a rationale for the study sample chosen. For studies involving existing datasets, please describe the dataset and source.*
Sampling strategy	*Describe the sampling procedure (e.g. random, snowball, stratified, convenience). Describe the statistical methods that were used to predetermine sample size OR if no sample-size calculation was performed, describe how sample sizes were chosen and provide a rationale for why these sample sizes are sufficient. For qualitative data, please indicate whether data saturation was considered, and what criteria were used to decide that no further sampling was needed.*
Data collection	*Provide details about the data collection procedure, including the instruments or devices used to record the data (e.g. pen and paper, computer, eye tracker, video or audio equipment) whether anyone was present besides the participant(s) and the researcher, and whether the researcher was blind to experimental condition and/or the study hypothesis during data collection.*
Timing	*Indicate the start and stop dates of data collection. If there is a gap between collection periods, state the dates for each sample cohort.*
Data exclusions	*If no data were excluded from the analyses, state so OR if data were excluded, provide the exact number of exclusions and the rationale behind them, indicating whether exclusion criteria were pre-established.*
Non-participation	*State how many participants dropped out/declined participation and the reason(s) given OR provide response rate OR state that no participants dropped out/declined participation.*
Randomization	*If participants were not allocated into experimental groups, state so OR describe how participants were allocated to groups, and if allocation was not random, describe how covariates were controlled.*

Ecological, evolutionary & environmental sciences study design

All studies must disclose on these points even when the disclosure is negative.

Study description	*Briefly describe the study. For quantitative data include treatment factors and interactions, design structure (e.g. factorial, nested, hierarchical), nature and number of experimental units and replicates.*
Research sample	*Describe the research sample (e.g. a group of tagged Passer domesticus, all Stenocereus thurberi within Organ Pipe Cactus National Monument), and provide a rationale for the sample choice. When relevant, describe the organism taxa, source, sex, age range and any manipulations. State what population the sample is meant to represent when applicable. For studies involving existing datasets, describe the data and its source.*
Sampling strategy	*Note the sampling procedure. Describe the statistical methods that were used to predetermine sample size OR if no sample-size calculation was performed, describe how sample sizes were chosen and provide a rationale for why these sample sizes are sufficient.*
Data collection	*Describe the data collection procedure, including who recorded the data and how.*
Timing and spatial scale	*Indicate the start and stop dates of data collection, noting the frequency and periodicity of sampling and providing a rationale for these choices. If there is a gap between collection periods, state the dates for each sample cohort. Specify the spatial scale from which the data are taken*
Data exclusions	*If no data were excluded from the analyses, state so OR if data were excluded, describe the exclusions and the rationale behind them, indicating whether exclusion criteria were pre-established.*
Reproducibility	*Describe the measures taken to verify the reproducibility of experimental findings. For each experiment, note whether any attempts to repeat the experiment failed OR state that all attempts to repeat the experiment were successful.*
Randomization	*Describe how samples/organisms/participants were allocated into groups. If allocation was not random, describe how covariates were controlled. If this is not relevant to your study, explain why.*

Blinding | *Describe the extent of blinding used during data acquisition and analysis. If blinding was not possible, describe why OR explain why blinding was not relevant to your study.*

Did the study involve field work? ☐ Yes ☒ No

Reporting for specific materials, systems and methods

We require information from authors about some types of materials, experimental systems and methods used in many studies. Here, indicate whether each material, system or method listed is relevant to your study. If you are not sure if a list item applies to your research, read the appropriate section before selecting a response.

Materials & experimental systems

n/a	Involved in the study	
☐	☒	Antibodies
☐	☒	Eukaryotic cell lines
☒	☐	Palaeontology and archaeology
☒	☐	Animals and other organisms
☒	☐	Clinical data
☒	☐	Dual use research of concern
☒	☐	Plants

Methods

n/a	Involved in the study	
☒	☐	ChIP-seq
☐	☒	Flow cytometry
☒	☐	MRI-based neuroimaging

Antibodies

Antibodies used | CD3-BUV395 (clone: SK7, supplier: BD Biosciences, catalogue number: 564001); CD19-BV421 (clone: HIB19, supplier: BD Biosciences catalogue number: 562440); CD5-FITC (clone:UCHT2, supplier: BD Biosciences, catalogue number: 555352); CD11b-PE (clone: ICRF44, supplier: eBioscience, catalogue number: 12-0118-42); CD33-BV510 (clone: WM53, supplier: BD Biosciences, catalogue number: 563257)

Validation | All antibodies used have had reactivity against human immunogen confirmed during quality control by the manufacturer and this is stated on the manufacturer's website. These clones have also been used in several published studies. Example references for each clone can be found below:
CD3 - van Dongen et al. (1988) Cytoplasmic Expression of the CD3 Antigen as a Diagnostic Marker for Immature T-Cell Malignancies. Blood 71:3.
CD19 - Caulier et al. (2024) CD37 is a safe chimeric antigen receptor target to treat acute myeloid leukemia. Cell Reports Medicine 5:6
CD5 - Li et al. (2017) Targeted Disruption of TCF12 Reveals HEB as Essential in Human Mesodermal Specification and Hematopoiesis. Stem Cell Reports 9:3
CD11b - Nicosia et al. (2022) Pharmacological inhibition of LSD1 triggers myeloid differentiation by targeting GSE1 oncogenic functions in AML. Oncogene 41
CD33 - Kyttälä et al. (2016) Genetic Variability Overrides the Impact of Parental Cell Type and Determines iPSC Differentiation Potential. Stem Cell Reports 6:2

Eukaryotic cell lines

Policy information about cell lines and Sex and Gender in Research

Cell line source(s) | The cell lines used in this study were K562 (human, female, ATCC CCL-243), HEK293-FT (human, female, ATCC CRL-1573) and OCI-AML2 (human, male, DSMZ ACC 99)

Authentication | None of the cell lines used were authenticated in this study.

Mycoplasma contamination | All cell lines used in the study were routinely tested for mycoplasma contamination and were negative.

Commonly misidentified lines (See ICLAC register) | No commonly misidentified cell lines were used in the study

Palaeontology and Archaeology

Specimen provenance | *Provide provenance information for specimens and describe permits that were obtained for the work (including the name of the issuing authority, the date of issue, and any identifying information). Permits should encompass collection and, where applicable, export.*

Specimen deposition | *Indicate where the specimens have been deposited to permit free access by other researchers.*

Dating methods | *If new dates are provided, describe how they were obtained (e.g. collection, storage, sample pretreatment and measurement), where*

Dating methods	*they were obtained (i.e. lab name), the calibration program and the protocol for quality assurance OR state that no new dates are provided.*

☐ Tick this box to confirm that the raw and calibrated dates are available in the paper or in Supplementary Information.

Ethics oversight	*Identify the organization(s) that approved or provided guidance on the study protocol, OR state that no ethical approval or guidance was required and explain why not.*

Note that full information on the approval of the study protocol must also be provided in the manuscript.

Animals and other research organisms

Policy information about studies involving animals; ARRIVE guidelines recommended for reporting animal research, and Sex and Gender in Research

Laboratory animals	*For laboratory animals, report species, strain and age OR state that the study did not involve laboratory animals.*
Wild animals	*Provide details on animals observed in or captured in the field; report species and age where possible. Describe how animals were caught and transported and what happened to captive animals after the study (if killed, explain why and describe method; if released, say where and when) OR state that the study did not involve wild animals.*
Reporting on sex	*Indicate if findings apply to only one sex; describe whether sex was considered in study design, methods used for assigning sex. Provide data disaggregated for sex where this information has been collected in the source data as appropriate; provide overall numbers in this Reporting Summary. Please state if this information has not been collected. Report sex-based analyses where performed, justify reasons for lack of sex-based analysis.*
Field-collected samples	*For laboratory work with field-collected samples, describe all relevant parameters such as housing, maintenance, temperature, photoperiod and end-of-experiment protocol OR state that the study did not involve samples collected from the field.*
Ethics oversight	*Identify the organization(s) that approved or provided guidance on the study protocol, OR state that no ethical approval or guidance was required and explain why not.*

Note that full information on the approval of the study protocol must also be provided in the manuscript.

Clinical data

Policy information about clinical studies

All manuscripts should comply with the ICMJE guidelines for publication of clinical research and a completed CONSORT checklist must be included with all submissions.

Clinical trial registration	*Provide the trial registration number from ClinicalTrials.gov or an equivalent agency.*
Study protocol	*Note where the full trial protocol can be accessed OR if not available, explain why.*
Data collection	*Describe the settings and locales of data collection, noting the time periods of recruitment and data collection.*
Outcomes	*Describe how you pre-defined primary and secondary outcome measures and how you assessed these measures.*

Dual use research of concern

Policy information about dual use research of concern

Hazards

Could the accidental, deliberate or reckless misuse of agents or technologies generated in the work, or the application of information presented in the manuscript, pose a threat to:

No	Yes	
☐	☐	Public health
☐	☐	National security
☐	☐	Crops and/or livestock
☐	☐	Ecosystems
☐	☐	Any other significant area

Experiments of concern

Does the work involve any of these experiments of concern:

No	Yes	
☐	☐	Demonstrate how to render a vaccine ineffective
☐	☐	Confer resistance to therapeutically useful antibiotics or antiviral agents
☐	☐	Enhance the virulence of a pathogen or render a nonpathogen virulent
☐	☐	Increase transmissibility of a pathogen
☐	☐	Alter the host range of a pathogen
☐	☐	Enable evasion of diagnostic/detection modalities
☐	☐	Enable the weaponization of a biological agent or toxin
☐	☐	Any other potentially harmful combination of experiments and agents

Plants

Seed stocks	*Report on the source of all seed stocks or other plant material used. If applicable, state the seed stock centre and catalogue number. If plant specimens were collected from the field, describe the collection location, date and sampling procedures.*
Novel plant genotypes	*Describe the methods by which all novel plant genotypes were produced. This includes those generated by transgenic approaches, gene editing, chemical/radiation-based mutagenesis and hybridization. For transgenic lines, describe the transformation method, the number of independent lines analyzed and the generation upon which experiments were performed. For gene-edited lines, describe the editor used, the endogenous sequence targeted for editing, the targeting guide RNA sequence (if applicable) and how the editor was applied.*
Authentication	*Describe any authentication procedures for each seed stock used or novel genotype generated. Describe any experiments used to assess the effect of a mutation and, where applicable, how potential secondary effects (e.g. second site T-DNA insertions, mosiacism, off-target gene editing) were examined.*

ChIP-seq

Data deposition

☐ Confirm that both raw and final processed data have been deposited in a public database such as GEO.

☐ Confirm that you have deposited or provided access to graph files (e.g. BED files) for the called peaks.

Data access links<br>*May remain private before publication.*	*For "Initial submission" or "Revised version" documents, provide reviewer access links. For your "Final submission" document, provide a link to the deposited data.*
Files in database submission	*Provide a list of all files available in the database submission.*
Genome browser session<br>(e.g. UCSC)	*Provide a link to an anonymized genome browser session for "Initial submission" and "Revised version" documents only, to enable peer review. Write "no longer applicable" for "Final submission" documents.*

Methodology

Replicates	*Describe the experimental replicates, specifying number, type and replicate agreement.*
Sequencing depth	*Describe the sequencing depth for each experiment, providing the total number of reads, uniquely mapped reads, length of reads and whether they were paired- or single-end.*
Antibodies	*Describe the antibodies used for the ChIP-seq experiments; as applicable, provide supplier name, catalog number, clone name, and lot number.*
Peak calling parameters	*Specify the command line program and parameters used for read mapping and peak calling, including the ChIP, control and index files used.*
Data quality	*Describe the methods used to ensure data quality in full detail, including how many peaks are at FDR 5% and above 5-fold enrichment.*
Software	*Describe the software used to collect and analyze the ChIP-seq data. For custom code that has been deposited into a community repository, provide accession details.*

Flow Cytometry

Plots

Confirm that:

☒ The axis labels state the marker and fluorochrome used (e.g. CD4-FITC).

☒ The axis scales are clearly visible. Include numbers along axes only for bottom left plot of group (a 'group' is an analysis of identical markers).

☒ All plots are contour plots with outliers or pseudocolor plots.

☒ A numerical value for number of cells or percentage (with statistics) is provided.

Methodology

Sample preparation	Cryopreserved mononuclear cells (MNCs) were thawed and washed twice in warmed RPMI supplemented with 10% FBS. For cell lines, cells were maintained in culture and transferred to a separate tube for downstream processing. Cells were centrifuged at 300g for 5 minutes and resuspended in FACS buffer (PBS supplemented with 0.1% BSA (Fisher BP9702-100)). Cells were counted and 1-3x106 cells were aliquoted into 1.5mL tubes. Cells were centrifuged at 300g for 5 minutes and resuspended in 1mL PBS containing 1:1000 Fixable Viability Dye eFluor™ 780 (eBioscience 65-0865-14) and incubated at 4°C in the dark for 20 minutes. Following this, cells were washed twice in FACS buffer. For the CLL sample only, cells were centrifuged at 300g for 5 minutes and resuspended in FACS buffer supplemented with the following antibodies: 1:100 CD3-BUV395, 1:160 CD19-BV421, 1:160 CD11b-PE, 1:100 CD33-BV510, 1:50 CD5-FITC (Supplementary Table 8). Cells were incubated at 4°C in the dark for 20 minutes and washed twice with FACS buffer and sorted as described below.<br>Following sorting (CLL sample) or viability staining (remaining samples), cells were centrifuged at 300g for 5 minutes at resuspended in 250μL of hybridization buffer (70% formamide (Thermo Scientific 17899), 20mM Tris (Thermo Scientific AM9850G) and 0.1% BSA in water) containing 0.3μg/mL TelC-Alexa647 (PNA Bio F1013) and 0.3μg/mL CENPB-Alexa488 (PNA Bio F3004) PNA probes which had been briefly heated at 55°C for 5 minutes and vortexed prior to addition. Cells were heated at 80°C for 10 minutes and incubated overnight at room temperature in the dark.<br>The following morning, cells were centrifuged at 300g for 7 minutes at 16°C and gently resuspended in 1mL of formamide wash buffer (70% formamide, 10mM Tris, 0.1% Tween 20 (Sigma P1379) and 0.1% BSA in water). This step was repeated once more. After this, cells were centrifuged at 300g for 7 minutes at 16°C and gently resuspended in 1mL of PBS wash buffer (PBS supplemented with 0.1% Tween 20 and 0.1% BSA). Finally, cells were centrifuged at 300g for 5 minutes at 16°C, resuspended in 500mL of FACS buffer supplemented with 10mg/mL RNase A (Invitrogen 12091021) and transferred to FACS tubes through a 40μm cell strainer (Fisher 22363547). Cells were sorted using a BD FACSAria Fusion flow cytometer.
Instrument	BD FACSAria Fusion
Software	Data was collected using BD FACSDiva software (v9.0) and analyzed in FlowJo (v10.10.0)
Cell population abundance	Cell population abundance was not determined post-sort. For telomere flow-FISH sorting, telomere qPCR was used to validate differences in telomere length between telomere-low and telomere-high groups
Gating strategy	For sorting of patient samples outlined in figure 4, the gating strategy is shown in Extended Data Fig. 6. Briefly, cells were gated using forward scatter (FSC) and side scatter (SSC). FSC-area and FSC-width were used to gate single cells. Following this, live cells were gated as Fixable Viability Dye-negative cells. In the CLL sample, additional gatings were used to first isolate CD5+ cells then CD19+CD3- cells. All samples were then gated to select cells in G1 using centromere probe then sorted by percentile telomere length. A small portion of the sample was used to establish the distribution of telomere lengths and then gates were set to sort cells in the following percentile telomere length ranges: <10%, 10-33%, 66-90%, >90%. In the CLL case, a more limited number of cells allowed only two gates to be sorted (<33%, >66%).

☒ Tick this box to confirm that a figure exemplifying the gating strategy is provided in the Supplementary Information.

Magnetic resonance imaging

Experimental design

Design type	*Indicate task or resting state; event-related or block design.*
Design specifications	*Specify the number of blocks, trials or experimental units per session and/or subject, and specify the length of each trial or block (if trials are blocked) and interval between trials.*
Behavioral performance measures	*State number and/or type of variables recorded (e.g. correct button press, response time) and what statistics were used to establish that the subjects were performing the task as expected (e.g. mean, range, and/or standard deviation across subjects).*

Acquisition

Imaging type(s) | *Specify: functional, structural, diffusion, perfusion.*

Field strength | *Specify in Tesla*

Sequence & imaging parameters | *Specify the pulse sequence type (gradient echo, spin echo, etc.), imaging type (EPI, spiral, etc.), field of view, matrix size, slice thickness, orientation and TE/TR/flip angle.*

Area of acquisition | *State whether a whole brain scan was used OR define the area of acquisition, describing how the region was determined.*

Diffusion MRI ☐ Used ☐ Not used

Preprocessing

Preprocessing software | *Provide detail on software version and revision number and on specific parameters (model/functions, brain extraction, segmentation, smoothing kernel size, etc.).*

Normalization | *If data were normalized/standardized, describe the approach(es): specify linear or non-linear and define image types used for transformation OR indicate that data were not normalized and explain rationale for lack of normalization.*

Normalization template | *Describe the template used for normalization/transformation, specifying subject space or group standardized space (e.g. original Talairach, MNI305, ICBM152) OR indicate that the data were not normalized.*

Noise and artifact removal | *Describe your procedure(s) for artifact and structured noise removal, specifying motion parameters, tissue signals and physiological signals (heart rate, respiration).*

Volume censoring | *Define your software and/or method and criteria for volume censoring, and state the extent of such censoring.*

Statistical modeling & inference

Model type and settings | *Specify type (mass univariate, multivariate, RSA, predictive, etc.) and describe essential details of the model at the first and second levels (e.g. fixed, random or mixed effects; drift or auto-correlation).*

Effect(s) tested | *Define precise effect in terms of the task or stimulus conditions instead of psychological concepts and indicate whether ANOVA or factorial designs were used.*

Specify type of analysis: ☐ Whole brain ☐ ROI-based ☐ Both

Statistic type for inference | *Specify voxel-wise or cluster-wise and report all relevant parameters for cluster-wise methods.*

(See Eklund et al. 2016)

Correction | *Describe the type of correction and how it is obtained for multiple comparisons (e.g. FWE, FDR, permutation or Monte Carlo).*

Models & analysis

n/a	Involved in the study
☐	☐ Functional and/or effective connectivity
☐	☐ Graph analysis
☐	☐ Multivariate modeling or predictive analysis

Functional and/or effective connectivity | *Report the measures of dependence used and the model details (e.g. Pearson correlation, partial correlation, mutual information).*

Graph analysis | *Report the dependent variable and connectivity measure, specifying weighted graph or binarized graph, subject- or group-level, and the global and/or node summaries used (e.g. clustering coefficient, efficiency, etc.).*

Multivariate modeling and predictive analysis | *Specify independent variables, features extraction and dimension reduction, model, training and evaluation metrics.*
